# Pressure and Flow Relations in the Systemic Arterial Tree Throughout Development From Newborn to Adult

**DOI:** 10.3389/fped.2020.00251

**Published:** 2020-05-19

**Authors:** Berend E. Westerhof, Martin J. C. van Gemert, Jeroen P. van den Wijngaard

**Affiliations:** ^1^Cardiovascular and Respiratory Physiology, Technical Medical Centre, Faculty of Science and Technology, University of Twente, Enschede, Netherlands; ^2^Pulmonary Medicine, Amsterdam Cardiovascular Sciences, Amsterdam UMC, Free Universiteit Amsterdam, Amsterdam, Netherlands; ^3^Medical Biology, Section of Systems Physiology, Amsterdam Cardiovascular Sciences, Amsterdam UMC, University of Amsterdam, Amsterdam, Netherlands; ^4^Biomedical Engineering and Physics, Amsterdam UMC, University of Amsterdam, Amsterdam, Netherlands; ^5^Clinical Chemistry Hematology and Immunology, Diakonessenhuis Utrecht, Utrecht, Netherlands

**Keywords:** distributed arterial model, newborn to adult, aortic, peripheral, blood pressure, flow, windkessel

## Abstract

**Objective:** Distributed models of the arterial tree allow studying the effect of physiological and pathophysiological changes in the vasculature on hemodynamics. For the adult, several models exist; however, a model encompassing the full age range from newborn to adult was until now lacking. Our goal is to describe a complete distributed hemodynamic model for normal development from newborn to adult.

**Methods:** The arterial system was modeled by 121 segments characterized by length, radius, wall thickness, wall stiffness, and wall viscosity. The final segments ended in three-element Windkessels. All parameters were adapted based on body height and weight as a function of age as described in the literature.

**Results:** Pressures and flows are calculated as a function of age at sites along the arterial tree. Central to peripheral transfer functions are given. Our results indicate that peripheral pressure in younger children resembles central pressure. Furthermore, total arterial compliance, inertance and impedance are calculated. Findings indicate that the arterial tree can be simulated by using a three-element Windkessel system. Pulse wave velocity in the aorta was found to increase during development.

**Conclusions:** The arterial system, modeled from newborn to adult bears clinical significance, both for the interpretation of peripheral measured pressure in younger and older children, and for using a Windkessel model to determine flow from pressure measurements.

## Introduction

Arterial models help in understanding of hemodynamics and allow simulated experimental interventions that may not be easily performed in the human (1). To date, full-scale distributed models describe the adult circulation (2–7); an overview is given by Reymond et al. (8). Descriptions of fetal (9, 10), neonatal (11), and infant (12) circulations do exist, however, usually with simplified arterial networks. Nevertheless, these models demonstrated clinical relevance.

Our general aim was to develop a full-scale distributed model of the systemic arterial tree, consisting of realistic segments derived from physiological measurements, throughout development from newborn to adult. To describe all model parameters as a function of age, growth charts were used. We investigated pressure-flow relations in the model, with as a first aim to determine the central to peripheral pressure transfer functions (describing the changes in the pressure wave shape that occur while the wave travels in the arterial system). The second aim was to derive and determine the applicability of Windkessel models, based on characteristic impedance and the lumped parameters, as found by network analysis, describing total arterial resistance, compliance, and inertance. The first aim provides understanding of the relation between peripheral measured pressure and central pressure. The second aim gives insight in the applicability of 3- or 4-element Windkessels for realistic modeling of the input impedance of systemic arterial tree (13). Combined, these aims may aid in calculating cardiac output from peripherally measured pressures.

## Methods

The model of the arterial tree was developed using the description of Westerhof (2) with the inclusion of three element Windkessel impedances at the end of the final segments as given by Stergiopulos (4). In total, the Westerhof model incorporates 121 segments, of which length, radius, vascular wall thickness, wall stiffness and viscoelastic properties are given (2, 14).

To model the development of the arterial system, the following age dependent descriptions were assembled: height; proportional length and radius of body divisions (e.g., head, thorax, abdomen, legs); weight; and based on the previous, proportional volume of body divisions. These descriptions allow determining the dimensions of the arterial segments for any age. Once these dimensions are known, the pressure and flow transfer function of each segment can be calculated. Bringing all pressure and flow transfer functions together, the entire arterial system can be modeled. A flow wave with the correct stroke volume and heart rate for each age is used as input. Then pressure and flow can be calculated in each segment.

Thus, the current model is based on two main constituents: first, the original model by Westerhof et al. (2) (some parameter changes to the original model will be detailed); and second, descriptions of body development (which will also be given explicitly).

### Normal Development From Newborn to Adult

**Figure 1:**
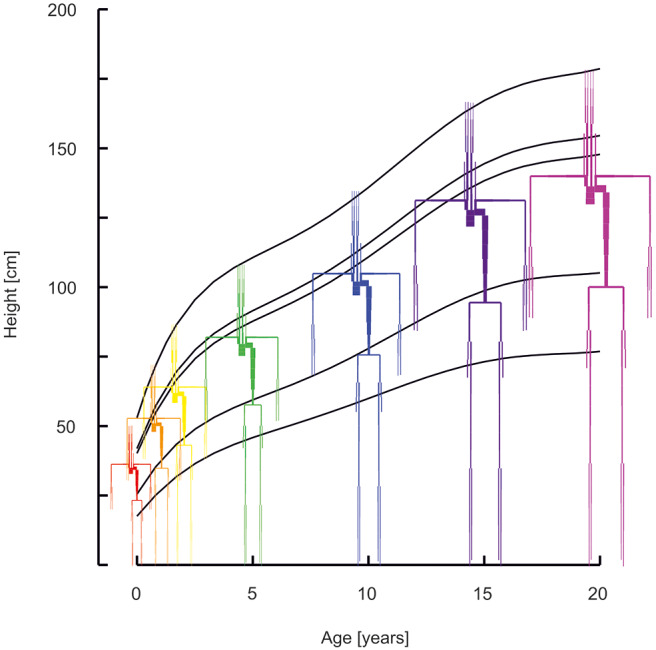
Arterial segments with the lengths and diameters as a function of age (red 0 years, orange 1 year, yellow 2 years, green 5 years, blue 10 years, purple 15 years, violet 20 years). Curves describe the development of stature, of the head, the neck, the thorax, the abdomen, and the legs.

The middle percentiles of the curves describing development as a function of age were used. Standard curves for stature as a function of age for boys were obtained from Freeman et al. (15). Curves for weight as a function of age were obtained from Wright et al. (16) (age 0–1 year) and Burmaster and Crouch (17) (age > 1 year). Growth was modeled separately for 6 divisions of the body: i.e., the head, neck, thorax, abdomen, legs, and arms. See Figure 1 for the age dependent development of stature and of the proportional development of body divisions. Using data on the relative dimensions of these body parts with respect to total body stature as a function of age, body divisions lengths and volumes were obtained (18). All body divisions were modeled by conical shapes, except for the head which was modeled by a sphere. Thus, all body divisions are essentially described by high order polynomials to yield dimensions as a function of age (Supplemental Material—Descriptions of body development).

### Vascular Wall Properties

To correctly model growth of the arterial system, i.e., segment length, radius and wall thickness, the following was considered. In the newborn, average tissue metabolism and tissue perfusion (19) are higher when compared to the adult as demonstrated by the larger ratio of cardiac output and body weight for the newborn vs. the adult (20, 21). Since tissue perfusion decreases with age, mean resistance to flow is increased with age. This implies that mean flow through the conduit arteries is relatively enhanced at younger age. Shear stress on the intimal layer (endothelium) is proportional to flow increase and inversely proportional to radial increase to the third power (22). Wall shear stress measurements at various ages show a relatively small or even absent dependence on age (23). Therefore, vessel radii of younger individuals were corrected to achieve normalized shear stress. In the model, this was implemented as follows; an increase in tissue perfusion at young age was accompanied by an increase in the vascular radius to a 1/3rd power so that shear stress remained within the normal range. As an example, if the flow to a certain body division of the adult would be 50 mL/min to 1 Kg of tissue, perfusion can be quantified as 50 (mL/min)/Kg. If tissue perfusion is higher at a younger age, e.g., 60 (mL/min)/Kg, then the radii of the conduit arteries supplying blood to this tissue are increased by a factor (60/50)^(1/3)^ = 1.06. Thus, a 20 % higher perfusion is accompanied by a 6 % increase in radius.

To account for an increased mean arterial pressure with increased age (24), vascular wall thickness was assumed to increase proportionally. Increased vascular wall thickness is a normal physiological response to increased mean arterial pressure and thereby normalizes vascular wall stress (9, 23–27). Elastance (Young's modulus) was not changed as a function of age.

### Transfer of Pressure and Flow

Transfer of pressure and flow over each segment is governed by transverse and longitudinal impedances. Transverse impedance is given by Jager et al. (28), longitudinal impedance is given by Womersley's theory of oscillatory flow for a longitudinally constrained tube (29). Both impedances can be obtained from vascular properties (22, 30). Computational details can be found elsewhere (1, 2, 31).

Starting from the distal impedances of the segment considered, pressure and flow transfer can be used to yield the impedance at the proximal site. Using standard circuit analysis for each frequency, multiple segments can be combined and finally resulting in the impedance of the entire arterial tree. Details on this approach can be found in the literature (1, 9, 10, 32, 33).

### Changes to the Original Model

The following adaptations were made to the original description of the arterial segments and wall properties (2). Radii of proximal aortic segments were adapted to remove sudden decrease in radius (see Supplemental Material: Vascular dimensions and Windkessel parameters: FIGURE Vascular dimensions—changes in the aortic radii). With these changes, the radii are close to those reported by Hickson et al. (34) for 20 years old individuals. In addition, the resistances of the abdominal vascular beds and of the brain were increased to reduce flows to a normal physiological levels (35) (see Supplemental Material: Vascular dimensions and Windkessel parameters: Table S1—Windkessel peripheral resistance increase with respect to the original model). Elastance of the carotid arteries was given values of the central- as opposed to the peripheral arteries. The elastance of all arterial segments (factor 0.5) and total peripheral resistances of all loading Windkessels (factor 0.75) were reduced since the model would otherwise generate hypertensive pressures (195/102 mmHg brachial pressure) for a normal stroke volume and cardiac output.

The parameters of the arterial vasculature for ages of 0, 1, 2, 5, 10, 15, and 20 years can be found in the Supplemental Material—Vascular dimensions and Windkessel parameters.

### Cardiac Output and Heart Rate

Cardiac output and heart rate were taken from Wiesener (21), where cardiac output was described to increase linearly from 564 mL/min at 0 to 5 L/min at 20 years and heart rate to decrease exponentially with weight to the power of −0.2 from 136 at 0 to 73 beats/min at 20 years (21, 22). The flow wave shape was chosen as a modified triangle, approaching a measured flow wave of which the maximum flow could be adapted to give the correct stroke volume. Ejection time was established as a function of heart rate (36, 37). The flow wave shape was used as input to the entire model, resulting in pressures and flows for each arterial segment.

### Pulse Wave Velocity

For the aortic segments, the Moens-Korteweg pulse wave velocities were calculated (22). Carotid to femoral pulse wave velocity was calculated from the difference in arrival time of the pressure waves in the carotid and femoral artery and the distance between the two. Distance was determined by adding the lengths of the arterial segments from the aorta to the carotid artery and from aorta to femoral artery.

### Windkessel Compliance and Resistance

Windkessel compliances are assumed to increase proportional to the volume of the body division they are in (18). Conversely, Windkessel resistance was taken inversely proportional to volume (22). In addition, Windkessel resistance was corrected to reflect perfusion decrease with age, as described in the vascular wall properties paragraph. The Windkessel parameters of the arterial vasculature for ages of 0, 1, 2, 5, 10, 15, and 20 years are listed in the Supplemental Material—Vascular dimensions and Windkessel parameters.

## Results

**Figure 2:**
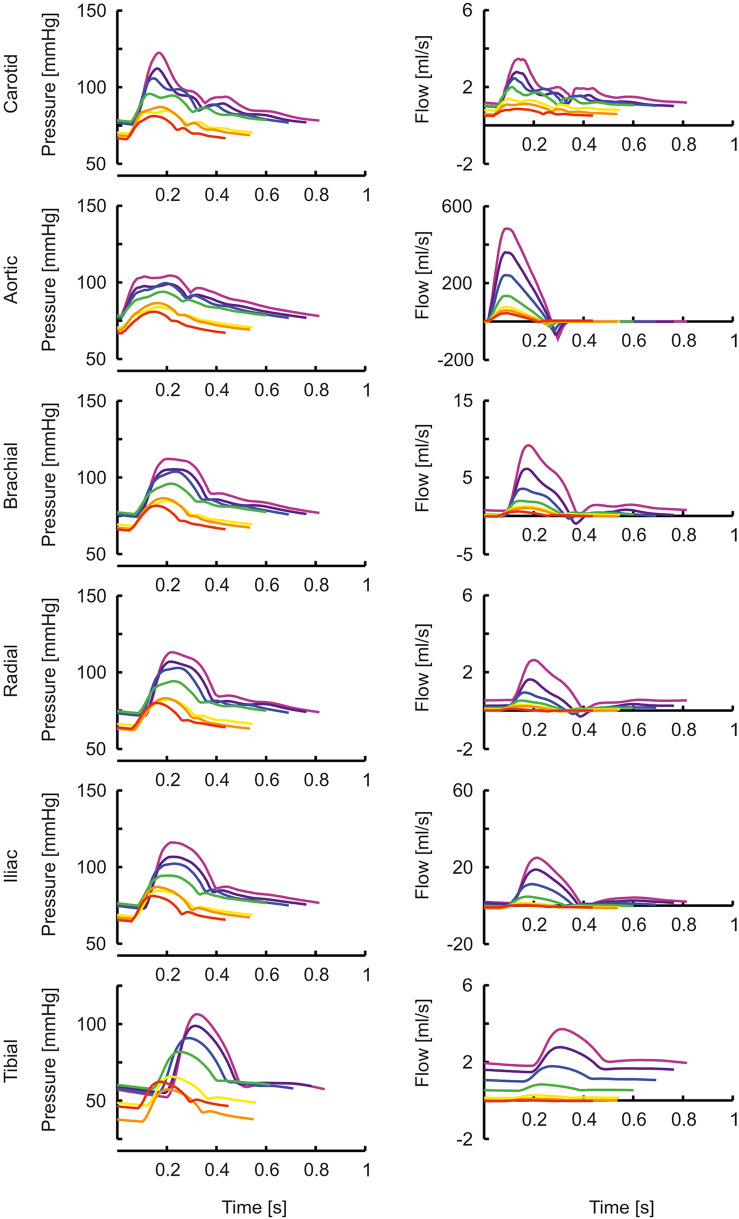
The pressure and flow waves for aorta, carotid, brachial, radial, femoral and tibial artery as a function of age (red, orange, yellow, green, blue, purple, violet: 0, 1, 2, 5, 10, 15, 20 years).

In Figure 1 the arterial segments are displayed with their lengths and diameters as a function of age. The pressure and flow waves are shown in Figure 2 for aorta, carotid, brachial, radial, femoral and tibial arteries. The pressure wave shapes are comparable for the age range between 0 and 5 years. Around the age of 10 the shapes start to change progressively. The ratio of brachial pulse pressure, respectively radial pulse pressure, to aortic pulse pressure, as measure of amplification, increased almost linearly from 1.15 to 1.39 (brachial) and from 1.19 to 1.55 (radial) over the years.

**Figure 3:**
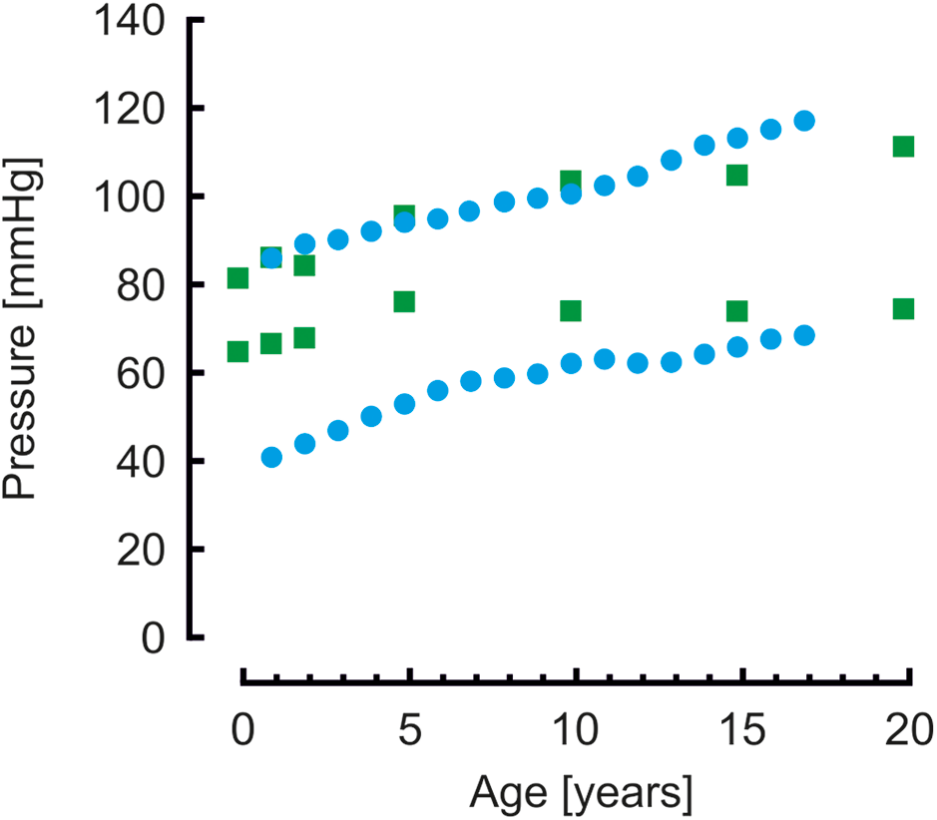
Brachial systolic and diastolic blood pressures from model simulations (green blocks) and from a population study (blue disks) (38). Systolic pressure are comparable, diastolic pressures of the model simulations are higher than the measured pressures.

Brachial systolic and diastolic blood pressures are presented in Figure 3. Pressures from a population study by Flynn et al. (38) in children aged 1–17 years are shown as reference. Systolic pressures of the model are similar whereas diastolic pressures are higher compared to measured pressures.

**Figure 4:**
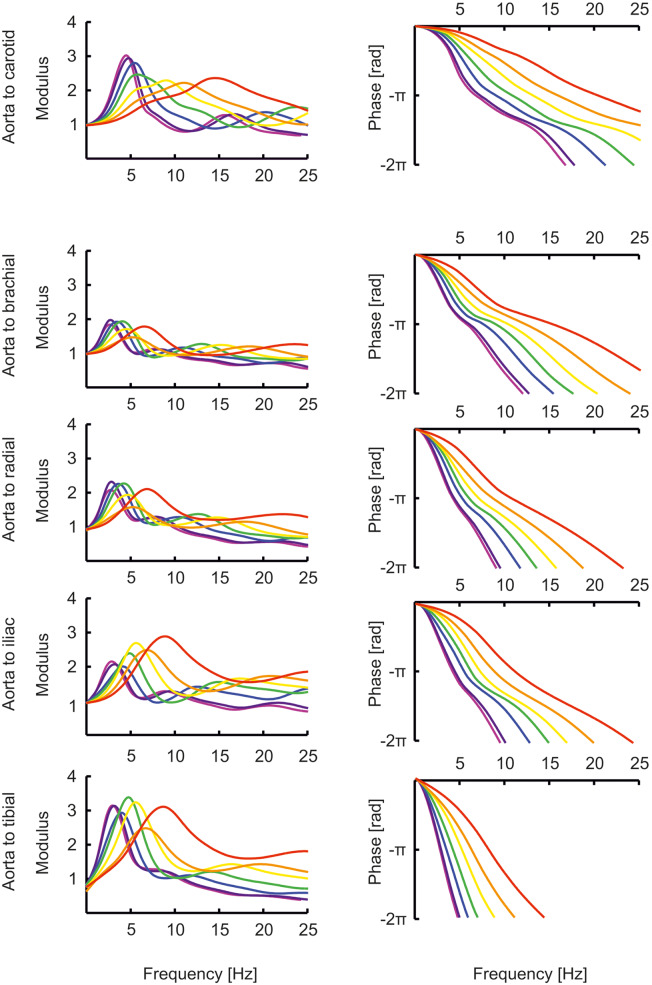
The transfer functions between aorta and the carotid, brachial, radial, femoral and tibial artery as a function of age (red, orange, yellow, green, blue, purple, violet: 0, 1, 2, 5, 10, 15, 20 years).

The transfer functions between aorta and the above-mentioned arteries are shown in Figure 4. In the “Supplemental Material: Transfer functions as function of harmonics” The transfer functions are plotted as a function of harmonics (instead of frequencies, Figure 4). By normalizing to the heart rate, a better insight is given in the effect of a transfer function for a certain age. In this representation, the peaks of the transfer functions are closer for the different ages than in Figure 4. However, the peaks of the younger ages are still at higher harmonics, explaining why their pressure wave shapes are less affected when traveling toward the periphery.

The percentage of resistance that resides in the vessels relative to the total resistance (thus including Windkessel resistance) was 11% at 0 years and decreased to 7% for 20 years, indicating a small pressure drop over the conduit arteries. When the shear rate was not kept constant by an increasing vascular radius to accommodate for the higher tissue perfusion at younger age, the resistance in the vessels relative to the total resistance was 26% at 0 years, causing a considerable pressure-drop.

**Figure 5:**
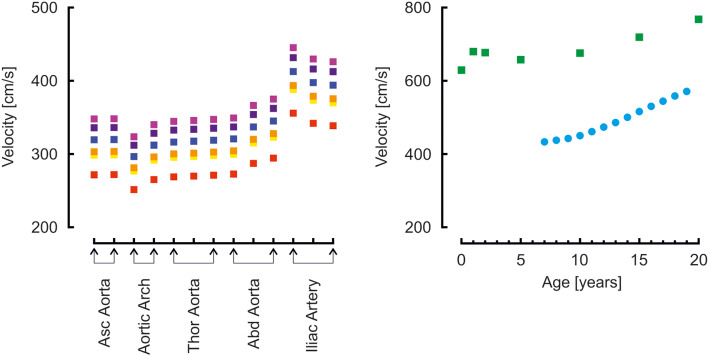
Left: Moens-Korteweg pulse wave velocity over the aortic segments as a function of age (red, orange, yellow, green, blue, purple, violet: 0, 1, 2, 5, 10, 15, 20 years). Right: carotid to femoral pulse wave velocity determined from model simulations (green blocks) and from a population study (blue disks) (39).

Moens-Korteweg pulse wave velocities (22) in the aortic segments and carotid to femoral pulse wave velocities are presented in Figure 5. For comparison, carotid to femoral pulse wave velocities reported by Reusz et al. (39) over ages ranging from 6 to 20 years are shown. Both model- and measured carotid to femoral velocities are higher than the Moens-Korteweg velocities. In all velocities an increase with age is visible.

Input impedances as determined from Fourier analysis of pressure and flow are given in the Supplemental Material—Vascular dimensions and Windkessel parameters, together with the 3-element (40) and 4-element (41) Windkessel impedances based on characteristic impedance and the lumping of the compliance, resistance and inertance of the distributed system (Table 1) (42). Both Windkessels fit to the data of the distributed system quite well.

**Table 1:** Lumped parameters of the arterial system.

**Age (years)**	**Z_**c**_ (g·cm^−4^·s^−1^)**	**L (g·cm^**−4**^)**	**C (g^−1^·cm^4^·s^2^)**	**R (g·cm^−4^·s^−1^)**
0	284	16.4	0.236	10,447
1	257	18.7	0.353	7,688
2	166	14.5	0.611	5,989
5	117	11.9	1.013	4,014
10	105	11.4	1.388	2,454
15	85	10.3	2.126	1,765
20	80	10.0	2.459	1,425

*Lumped parameters Z_c_ (characteristic impedance), L (inertance), C (compliance), and R (resistance) of the whole arterial system for ages 0–20 years*.

## Discussion

A model of the arterial system was developed that describes hemodynamics during development from 0 to 20 years, from newborn to adult. The system is based on anatomy, stature, proportional growth of different divisions of the body, and weight, all as described in the literature (15, 17, 18).

Our main findings are that the pressures for the age range of 0–5 years are quite similar throughout the arterial system. After the age of 10 years, the pressure transfer functions appear to have taken their final forms, remaining quite similar during further development to adulthood. This suggests that transfer functions developed for the adult may work acceptably for children of 10 years and older, but not for younger children. In the latter group, less amplification is present, and an adult transfer function would overcorrect systolic pressures, resulting in estimations that are too low. This observation fits with recent findings of systolic pressure underestimation is younger children (43, 44). Individualization of transfer functions based on body height or age may improve precision (32, 33).

Gevers et al. (45) used high-fidelity catheter tip-manometers to collect peripheral pressure (radial and tibial artery) waveforms in critically ill newborns. The radial pressures were very similar in form to central aortic pressures as seen in adults, many times with A-type (46) pressure augmentation visible. Tibial pressures were shown to resemble the more proximal femoral artery pressure in adult. The authors concluded that peripheral pressure in newborns have a central appearance.

The transfer functions derived by Cai et al. (47) give the relation between carotid (representing central pressure) and radial artery are not directly comparable to the transfer functions shown here which all give the relation between aortic pressure and peripheral pressures. Nonetheless, their transfer functions for pediatric (8 years old) and adolescent (14 years old) populations are similar to our aortic-to-radial transfer functions for 10 years and 15 years old [(47); Figures 2, 4]. The representation of their transfer functions is inverted compared to ours, and their troughs of about 0.5 correspond to our peaks of about 2. In addition, in their data, the trough in the transfer function deepens for higher age and moves to lower frequencies, analogous to the increasing peaks in our data, which move to lower frequencies as well.

Milne et al. (48) measured the carotid distension wave with echo-tracking and compared it with invasively measured aortic pressure. Also, in another population, they compared central pressure, reconstructed from non-invasively measured radial pressure using an adult transfer function, with the carotid distension wave. In both sub-studies the ages ranged between 2 and 18 years old; subjects were not stratified. The systolic pressure derived from the carotid distension wave (using the invasive diastolic and mean pressures for calibration) slightly overestimated the aortic pressure, but only some 4 mmHg. We find similar numbers for pressure augmentation in our study (1 mmHg for 5 years old, 6 mmHg for 10 years old, 13 mmHg for 15 years old). In the study of Milne et al. (48), the central systolic pressure reconstructed from radial pressure using the adult transfer function, and calibrated using auscultatory brachial pressures, was within 1 mmHg accurate when compared to the carotid distension wave. The central-to-peripheral pressure augmentation was almost 20 mmHg. This is contrary to our findings and to those of others (47) and may have to do with the excessive reduction of peripherally measured systolic pressure when using an adult transfer function in children.

In older age, it is quite well-established that pulse pressure amplification is reduced (49, 50), and brachial and radial systolic pressures are closer to central aortic systolic pressure than in younger age. Pulse pressure amplification is determined by the transfer function between the aorta and the brachial or radial site. In a 20 years old individual, the peak in the transfer function is at a relatively low frequency, so that the lower frequencies (which are the main determinants of the pressure levels) are amplified. At older age, when vessels become stiffer, the peak moves to higher frequencies, away from the main frequency content, and amplification is reduced (31, 51). Views on the effects of pulse pressure amplification in the younger ages are divided (52). The effects are relevant to make an appropriate decision when considering treatment of early age hypertension (53). Based on our modeling results, we suggest that pressure augmentation is smaller in younger individuals. We agree with Adji and O'Rourke (54) that the changes in vascular stiffness in this age group are small, and the changes in the transfer functions are determined mainly by changes in dimensions. We however would advise to be cautious with the use of an adult transfer function for the very young; probably, the results are acceptable from 10 years and up.

When comparing brachial pressures of the model to measured pressures in a population study by Flynn et al. (38), systolic pressures were similar, however, diastolic pressures of the model were higher, in particular for the younger ages. This suggests that the arterial stiffness of the model is too low, resulting in an overly low pulse pressure.

Our model is highly versatile and allows making changes to describe diverse anatomical aberrations (coarctation, aneurysm). It could also be used to investigate the effects of childhood obesity on the pressure wave shape (55, 56). Also potentially relevant is model based prediction of the effect of vascular remodeling, which may start already early in life in children with end-stage renal disease, chronic parenteral nutrition, HIV infection, or aortic coarctation, as suggested by Aggoun et al. (57).

Both the 3- as well as the 4-element Windkessel seem to provide an acceptable lumped parameter model description of the arterial input impedance. The parameters can be estimated based on gender (which we did not investigate), age, height and weight. We suggest that the findings of the current study can serve as a basis for further development of a Windkessel model, which could be used to estimate cardiac output from pressure measurements (58). To our knowledge, such a model is currently not available for children.

### Cardiac Output and Heart Rate

The cardiac output and heart rate taken from Wiesener (21) gave correct mean arterial pressures as a function of age (24), but only after the total peripheral resistance of the original adult model (2) was reduced. Also, the arterial stiffness of the original description (2) was reduced to arrive at acceptable pulse pressures. To check if the used cardiac output as a function of age is also reasonable, the following calculations were done. Body weight increased from 3.5 kg at birth to 78 kg at 20 years; cardiac output increased from 564 mL/min to 5 L/min. Dividing cardiac output by body weight gives 161 and 64 mL/min/kg, respectively. Thus, tissue perfusion is indeed higher in youth than at later ages, as expected (19). Alternatively, cardiac output has been suggested to be 933 × weight^0.38^ in children and adolescents (59). At 3.5 kg, the cardiac output then would be calculated as 1.5 L/min, or 430 mL/min/kg, which is almost seven times higher than the value in the adult circulation, which we believe is a quite unacceptably high tissue perfusion. The first estimation gives a factor of 3 higher tissue perfusion for children vs. adults, which to us seems more realistic.

Across the mammalian species, cardiac output is related to body weight^3/4^ (22). With weight increase from 3.5 to 78 kg, from the newborn to adult, a factor of 22, cardiac output should increase by a factor of 22^3/4^ = 10.2. This is in line with the cardiac output increase reported by Wiesener (21).

### Shear Stress

As previously established from non-invasive measurements (23), we assumed that shear stress in the conduit vessels during development from the newborn to the adult circulation, would remain constant (23). Cardiac output increased approximately a factor of 10, so that arterial radii should increase by a factor of 10^1/3^ or approximately 2.15 times: i.e., from around 0.68–1.47 cm for the aorta radius. If cardiac output was actually as high as 1.5 L/min (59) at 0 years (see section “Cardiac output and heart rate” in the Discussion), then the cardiac output would increase only about 3.33 instead of 10 times. With a radius increase of 3.33^1/3^ or approximately 1.49 times, aortic radius would be about 1 cm at 0 years, which is rather unrealistically large.

With the implementation of a constant wall shear stress, the resistance in the conduit vessels was low with respect to total resistance (including the resistance of the loading Windkessels), thus the pressure drop in the vessels was small for the given cardiac outputs. Without this implementation of constant wall shear stress, the pressure drop would have become substantial.

### Pulse Wave Velocity

Pulse wave velocity showed a gradual increase over the years. At younger age, vascular diameters were smaller, however, wall thickness was relatively even more reduced, since the mean arterial pressures were lower (see section “Vascular wall properties” in the Methods). This results in a lower wall thickness/radius ratio and thus, according to Moens-Korteweg (22), a lower pulse wave velocity as compared to the value at higher age.

Carotid to femoral velocities were higher than the aortic Moens-Korteweg velocities (22). In the study by Reusz et al. (39), the distance was measured over the body; in our model, segments lengths were added. In this way the travel distance is overestimated (this approach assumes that the pressure wave travels from the carotid to the femoral artery), resulting in an overestimation of aortic pulse wave velocity (60, 61). This is indeed apparent from the findings. The model findings overestimate Moens-Korteweg velocities even more than the measured data (39), likely since the addition of the segments lengths overestimates the measured lengths.

Another explanation is that arterial stiffness in the model is too high. To obtain pulse wave velocities at the same absolute value as Reusz et al. (39) found at 20 years of age, i.e., about 500 cm/s instead of the 700 cm/s found in the model, the vascular stiffness was reduced by a factor of 0.5. This gives a reduction of pulse wave velocity by a factor of 0.7. The aortic pressure then became 100/83 mmHg, brachial pressure 102/81. This is a very low pulse pressure and at odds with the measurements of by Flynn et al. (38), who report 117/68 at 17 years of age (121/74 when extrapolated to 20 years). Thus, the model strikes the balance: on the one hand, a higher arterial stiffness would give a pulse pressure closer to the reported measurements of Flynn et al. (38); on the other hand, a lower arterial stiffness would give carotid to femoral pulse wave velocities closed to those reported by Reusz et al. (39). More studies are needed to accurately determine arterial stiffness in developing children.

### Model Considerations

Our work is a first step in the development of a distributed model that accurately describes pressure and flow relations in healthy children. The length, radius and wall thickness of the arterial segments were estimated based on growth curves and not measured in groups of individuals in certain age ranges. Detailed measurements of the vasculature as a function of age would possibly allow further improvement of the model description. Potential sex differences that may include differences in cardiac output and tissue perfusion were not considered.

In the current model, the arterial stiffness of the arterial segments is based on the original description of Westerhof et al. (2) distinguishing only four separate values. In reality, the distribution of stiffness is probably gradual, with the distal arteries being stiffer (62). Moreover, these four stiffness values were kept the same during the 20-years development. Also, in the model, the stiffness of each vessel is taken constant while it actually depends on the pressure that extends the vessel wall. While this simplification may be acceptable in the normal situation, with a limited range of pressure levels, a more accurate non-linear description would enhance the applicability of the model, e.g., in severe hypotension.

Radii of the arterial segments were assumed to grow proportional to the increase of length of the body division. When keeping the shear rate constant during development, by correcting radii to accommodate for the higher tissue perfusion at younger age, the resistance of the conduit vessels was acceptably low relative to the tissue resistance for all ages. Without shear stress correction however the conduit vessel resistance would cause considerably lower pressures at tissue level at younger ages. Our first assumption that radii would increase with length may be incorrect, but, together with the shear stress correction, seems to give acceptable results.

The model describes the systemic arterial tree with a closed ductus arteriosus (and in the heart, the foramen ovale is supposed to be closed). Interesting future work could aim to describe the transition from the neonatal circulation toward the closing of these shunts and the impact on cardiac output (63).

In our approach, the flow wave shape was the same for all simulations. A different flow wave shape may give other pressure wave shapes and for instance give rise to higher systolic pressure amplification. However, since the transfer functions are only determined by arterial parameters, these are independent of the flow wave shape that was used.

Finally, reflexes of the autonomic nervous system and autoregulation of organs were not incorporated in the model.

## Conclusion

To our knowledge, we presented the currently most complete distributed model of the arterial system for the age range from newborn to adult. Important results encompass the finding of peripheral pressures in younger children to resemble their central pressure, and that input impedance may be described by a simple Windkessel model.

The results carry clinical significance for the interpretation of peripheral measured pressures in younger and older children. Further application of our model may e.g., aid in estimating cardiac output in children in intensive care, or to investigate effects resulting from vascular aberrations.

## Data Availability Statement

All datasets generated for this study are included in the article/Supplementary Material.

## Author's Note

Main findings of this study were presented at the Artery 2011 meeting.

## Author Contributions

BW and JW conceived the study, developed the model, and wrote the manuscript. MG wrote the manuscript.

## Conflict of Interest

The authors declare that the research was conducted in the absence of any commercial or financial relationships that could be construed as a potential conflict of interest.
