# Supplementary material for: Pressure and Flow Relations in the Systemic Arterial Tree Throughout Development From Newborn to Adult
Source: Front Pediatr. 2020 May 19;8:251. doi: 10.3389/fped.2020.00251 (PMC7248228; doi:10.3389/fped.2020.00251)
Supplement: Supplementary file 1 [file Data_Sheet_1.pdf]

Supplemental material:
Vascular dimensions and Windkessel parameters

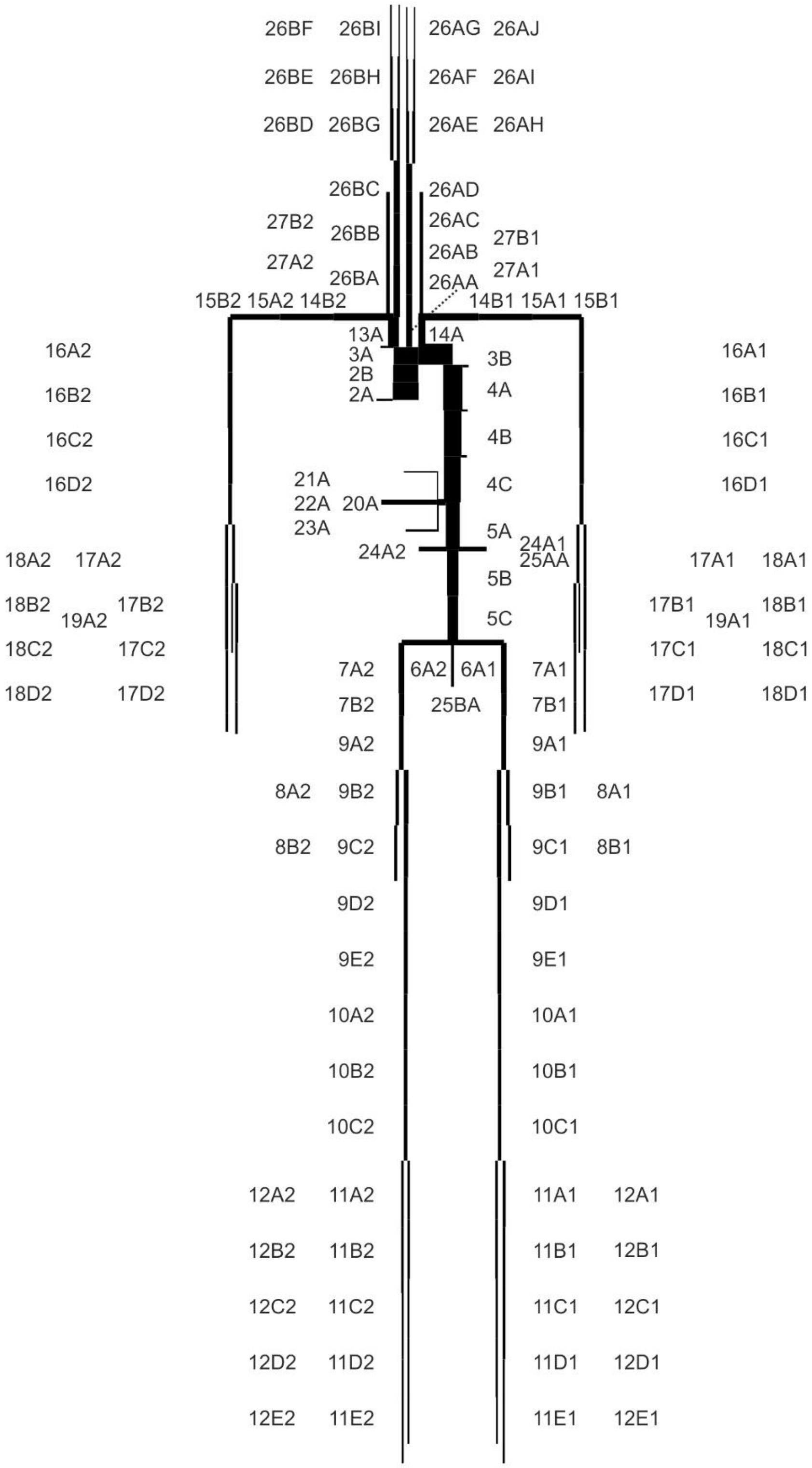

SUPPLEMENTAL FIGURE Vascular dimensions − identification of segments

Identification of the modelled arterial segments. Segments plotted to scale. For the names of the segments see the Tables of this Supplement.

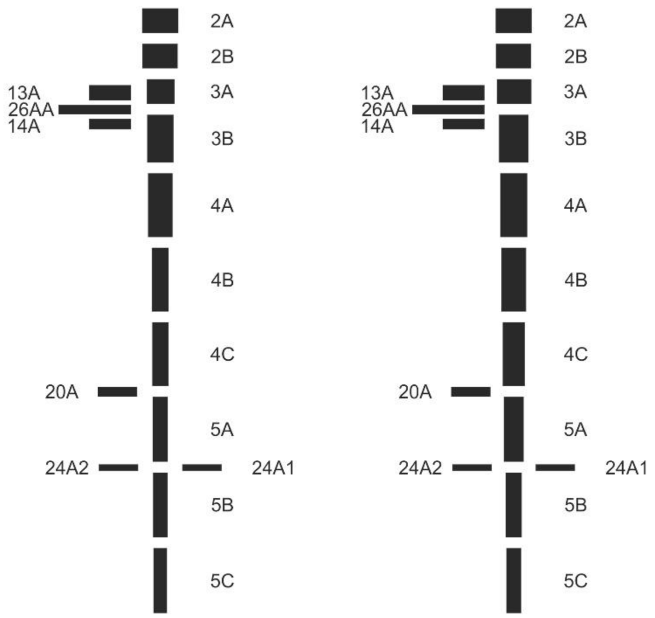

SUPPLEMENTAL FIGURE Vascular dimensions − changes in the aortic radii

Illustration of the changes made to the radii of the aortic segments of the original model. Left, aortic dimensions in the original description [1]. Right, the changed radii of the current model. In the original description, a sudden decrease between segment 2B and 3A and between 4A and 4B was noted. This is unexpected, since there are no major branches. In the model of the current study, the decrease of the radii was made more gradual. The radii in the right panel are close to those reported by Hickson et al. [2] for 20 year old individuals, except for the most distal segment, which is somewhat smaller. In our adult model, the diameters of segments 2B, 4A, 4C, 5A and 5C are 2.88, 2.2, 1.8, 1.6, 1.2 cm, while Hickson et al. [2] report 2.9, 2.1, 1.8, 1.7 and 1.6 cm.

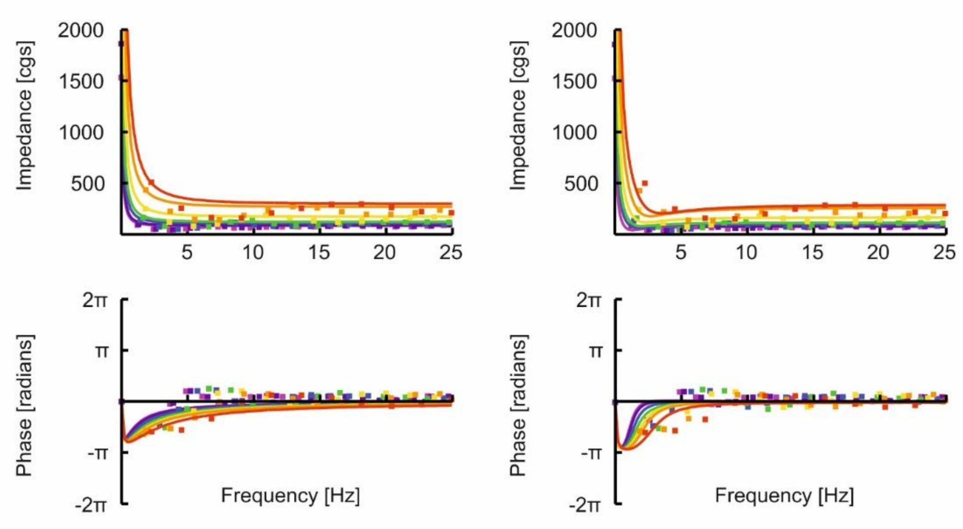

SUPPLEMENTAL FIGURE Input impedance and Windkessel impedance

Input impedances (boxes) and Windkessel impedances (drawn; left: 3-element Windkessel; right, 4-element Windkessel). Windkessel impedances are based on characteristic impedance and the lumping of the compliance, resistance and inertance (for the 4-element Windkessel; see **Table 1** in the main manuscript) of the distributed system as a function of age (red, orange, yellow, green, blue, purple, violet: 0, 1, 2, 5, 10, 15, 20 years).

Windkessel peripheral resistance increase		
		Factor
At the end of:	21A	1.5
	22A	1.5
	23A	1.5
	24A1&2	1.5
	25AA	1.5
	25BA	1.5
	26AG	1.25
	26AJ	1.25
	26BF	1.25
	26BI	1.25
	27B1&2	1.25

SUPPLEMENTAL TABLE S1 - Windkessel peripheral resistance increase with respect to the original model [1]. With the resistance increases listed here, for the adult, flow through the celiac (21A+22A+23A) and mesenteric arteries (25AA+25BA) is 25%; renal flow (24A1 and 24A2) is 22%; and flow to the brain (through segments 26AG+26AJ+26BF+26BI and 27B1&2) is 15% of total flow [3].

0 years					
**Arterial segments**		**Vascular dimensions**			
Notation of segment	Name of artery	length	radius	wall thickness	Young's modulus
2A	Aorta ascendens	0.687716	0.688217	0.0438610	2E+06
2B	Aorta ascendens	0.687716	0.674172	0.0430586	2E+06
3A	Arcus aorta	0.687716	0.655445	0.0353027	2E+06
3B	Arcus aorta	1.341046	0.561810	0.0339655	2E+06
4A	Aorta thoracalis	1.788061	0.514993	0.0320934	2E+06
4B	Aorta thoracalis	1.788061	0.468175	0.0294190	2E+06
4C	Aorta thoracalis	1.788061	0.421358	0.0267445	2E+06
5A	Aorta abdominalis	1.822447	0.374540	0.0240701	2E+06
5B	Aorta abdominalis	1.489823	0.248772	0.0179278	2E+06
5C	Aorta abdominalis	1.489823	0.229636	0.0174906	2E+06
6A	A. iliaca communis	1.630372	0.130127	0.0153043	2E+06
7A	A. iliaca externa	1.630372	0.122472	0.0131179	2E+06
7B	A. iliaca externa	0.702747	0.114818	0.0120248	2E+06
8A	A. profundus	1.439209	0.062207	0.0092394	8E+06
8B	A. profundus femoris	1.439209	0.055987	0.0081733	8E+06
9A	A. femoralis	1.714702	0.107163	0.0115875	2E+06
9B	A. femoralis	1.393520	0.080870	0.0092394	2E+06
9C	A. femoralis	1.393520	0.074649	0.0090617	2E+06
9D	A. femoralis	1.393520	0.071539	0.0088840	2E+06
9E	A. femoralis	1.621965	0.068428	0.0087063	2E+06
10A	A. poplitea	1.439209	0.065318	0.0085286	4E+06
10B	A. poplitea	1.439209	0.062207	0.0083510	4E+06
10C	A. poplitea	1.439209	0.059097	0.0081733	4E+06
11A	A. tibialis posterior	1.530587	0.049766	0.0090617	8E+06
11B	A. tibialis posterior	1.530587	0.046656	0.0087063	8E+06
11C	A. tibialis posterior	1.530587	0.043545	0.0081733	8E+06
11D	A. tibialis posterior	1.530587	0.040435	0.0078179	8E+06
11E	A. tibialis posterior	1.210763	0.037324	0.0072849	8E+06
12A	A. tibialis anterior	1.713344	0.049766	0.0069295	8E+06
12B	A. tibialis anterior	1.713344	0.046656	0.0069295	8E+06
12C	A. tibialis anterior	1.713344	0.043545	0.0069295	8E+06
12D	A. tibialis anterior	0.982317	0.037324	0.0069295	8E+06
12E	A. tibialis anterior	1.713344	0.040435	0.0069295	8E+06
13A	A. anonyma	1.169117	0.290269	0.0230003	2E+06
14A	A. subclavia	1.169117	0.198038	0.0179188	2E+06
14B	A. subclavia	2.168186	0.174953	0.0163677	2E+06
15A	A. axillaris	1.944990	0.158022	0.0153757	2E+06
15B	A. axillaris	1.785565	0.136316	0.0141357	2E+06
16A	A. brachialis	2.008760	0.122424	0.0136397	2E+06
16B	A. brachialis	2.008760	0.115478	0.0131437	2E+06
16C	A. brachialis	2.008760	0.108532	0.0128957	2E+06
16D	A. brachialis	1.466714	0.102454	0.0123998	2E+06
17A	A. ulnaris	2.136301	0.078143	0.0121518	4E+06
17B	A. ulnaris	2.136301	0.069460	0.0116558	4E+06
17C	A. ulnaris	2.136301	0.065119	0.0114078	4E+06
17D	A. ulnaris	1.179748	0.060778	0.0111598	4E+06
18A	A. radialis	2.263841	0.073802	0.0109118	4E+06
18B	A. radialis	2.263841	0.069460	0.0106638	4E+06
18C	A. radialis	2.263841	0.065119	0.0104158	4E+06
18D	A. radialis	0.701472	0.060778	0.0101678	4E+06
19A	A. interossea volaris	2.518922	0.039071	0.0069439	8E+06
20A	A. coelica	0.343858	0.182588	0.0171165	2E+06
21A	A. gastrica sin.	2.441391	0.084272	0.0120350	2E+06
22A	A. lienalis	2.166305	0.128748	0.0144420	2E+06
23A	A. hepatica	2.269462	0.102999	0.0131048	2E+06
24A	A. renalis	0.899516	0.099509	0.0113689	2E+06
25AA	A. mesenterica sup.	1.658482	0.076545	0.0150856	2E+06
25BA	A. mesenterica inf.	1.405493	0.061236	0.0094012	2E+06

26AA	A. carotis com. sin.	2.028762	0.173225	0.0168490	1E+06
26AB	A. carotis com. sin.	1.544946	0.131914	0.0128309	1E+06
26AC	A. carotis com. sin.	1.544946	0.131914	0.0128309	1E+06
26AD	A. carotis com. sin.	0.811751	0.131914	0.0128309	1E+06
26AE	A. carotis int. sin.	2.709658	0.110679	0.0160742	8E+06
26AF	A. carotis int. sin.	2.709658	0.080664	0.0139310	8E+06
26AG	A. cerebri anterior sin.	2.709658	0.051900	0.0092873	16E+06
26AH	A. carotis ext. sin.	2.709658	0.110679	0.0160742	8E+06
26AI	A. carotis ext. sin.	2.709658	0.080664	0.0139310	8E+06
26AJ	A. carotis ext. sin.	2.709658	0.051900	0.0092873	16E+06
26BA	A. carotis com. dextra.	1.544946	0.131914	0.0128309	1E+06
26BB	A. carotis com. dextra.	1.544946	0.131914	0.0128309	1E+06
26BC	A. carotis com. dextra.	1.544946	0.131914	0.0128309	1E+06
26BD	A. carotis ext. dextra.	2.709658	0.110679	0.0160742	8E+06
26BE	A. carotis ext. dextra.	2.709658	0.080664	0.0139310	8E+06
26BF	A. carotis ext. dextra.	2.709658	0.051900	0.0092873	16E+06
26BG	A. carotis int. dextra.	2.709658	0.110679	0.0160742	8E+06
26BH	A. carotis int. dextra.	2.709658	0.080664	0.0139310	8E+06
26BI	A. cerebri anterior dextra	2.709658	0.051900	0.0092873	16E+06
27A	A. vertebralis	1.859172	0.067027	0.0093686	2E+06
27B	A. vertebralis	1.859172	0.065244	0.0091649	2E+06

		**Windkessel parameters**		
		Rc	Rp	Cw
Before:	2A	167494	218157	2.68876E-07
Between:	3A and 3B	167494	1469851	1.02818E-06
	3B and 4A	167494	1469851	1.02818E-06
	4A and 4B	167494	1469851	1.02818E-06
	4B and 4C	167494	1469851	1.02818E-06
At the end of:	21A	28533	628942	2.64182E-07
	22A	11288	270375	6.16040E-07
	23A	18414	422389	3.93719E-07
	24A	18822	83989	1.99212E-06
	25AA	39033	68885	1.99212E-06
	25BA	54388	338901	3.27191E-07
	26AG	224160	257008	4.99049E-07
	26AJ	224160	257008	4.99049E-07
	26BF	224160	257008	4.99049E-07
	26BI	224160	257008	4.99049E-07
	27B	46451	102775	6.04114E-07
	8B	126513	704629	1.57242E-07
	11E	305106	704629	1.57242E-07
	12E	249744	825507	1.34174E-07
	17D	83747	770877	2.63490E-07
	18D	80753	1271549	2.63490E-07
	19A	270150	6715368	1.65034E-08

SUPPLEMENTAL TABLE S2 - Vascular dimensions and Windkessel parameters for 0 years of age.

1 year

Arterial segments		Vascular dimensions			
Notation of segment	Name of artery	length	radius	wall thickness	Young's modulus
2A	Aorta ascendens	0.962513	0.765220	0.0622639	2E+06
2B	Aorta ascendens	0.962513	0.749603	0.0611249	2E+06
3A	Arcus aorta	0.962513	0.728781	0.0501149	2E+06
3B	Arcus aorta	1.876901	0.624669	0.0482166	2E+06
4A	Aorta thoracalis	2.502534	0.572613	0.0455590	2E+06
4B	Aorta thoracalis	2.502534	0.520558	0.0417624	2E+06
4C	Aorta thoracalis	2.502534	0.468502	0.0379658	2E+06
5A	Aorta abdominalis	2.550660	0.416446	0.0341692	2E+06
5B	Aorta abdominalis	1.985035	0.263328	0.0242283	2E+06
5C	Aorta abdominalis	1.985035	0.243072	0.0236373	2E+06
6A	A. iliaca communis	2.172303	0.137741	0.0206827	2E+06
7A	A. iliaca externa	2.172303	0.129639	0.0177280	2E+06
7B	A. iliaca externa	0.936337	0.121536	0.0162507	2E+06
8A	A. profundus	2.188334	0.075144	0.0142493	8E+06
8B	A. profundus femoris	2.188334	0.067630	0.0126051	8E+06
9A	A. femoralis	2.284663	0.113434	0.0156597	2E+06
9B	A. femoralis	2.118863	0.097687	0.0142493	2E+06
9C	A. femoralis	2.118863	0.090173	0.0139752	2E+06
9D	A. femoralis	2.118863	0.086416	0.0137012	2E+06
9E	A. femoralis	2.466217	0.082659	0.0134272	2E+06
10A	A. poplitea	2.188334	0.078901	0.0131532	4E+06
10B	A. poplitea	2.188334	0.075144	0.0128791	4E+06
10C	A. poplitea	2.188334	0.071387	0.0126051	4E+06
11A	A. tibialis posterior	2.327276	0.060115	0.0139752	8E+06
11B	A. tibialis posterior	2.327276	0.056358	0.0134272	8E+06
11C	A. tibialis posterior	2.327276	0.052601	0.0126051	8E+06
11D	A. tibialis posterior	2.327276	0.048844	0.0120571	8E+06
11E	A. tibialis posterior	1.840979	0.045086	0.0112350	8E+06
12A	A. tibialis anterior	2.605159	0.060115	0.0106869	8E+06
12B	A. tibialis anterior	2.605159	0.056358	0.0106869	8E+06
12C	A. tibialis anterior	2.605159	0.052601	0.0106869	8E+06
12D	A. tibialis anterior	1.493625	0.045086	0.0106869	8E+06
12E	A. tibialis anterior	2.605159	0.048844	0.0106869	8E+06
13A	A. anonyma	1.636273	0.322746	0.0326506	2E+06
14A	A. subclavia	1.636273	0.220196	0.0254371	2E+06
14B	A. subclavia	2.983380	0.191248	0.0228433	2E+06
15A	A. axillaris	2.676267	0.172740	0.0214589	2E+06
15B	A. axillaris	2.456901	0.149012	0.0197283	2E+06
16A	A. brachialis	2.764014	0.133826	0.0190361	2E+06
16B	A. brachialis	2.764014	0.126233	0.0183439	2E+06
16C	A. brachialis	2.764014	0.118640	0.0179978	2E+06
16D	A. brachialis	2.018169	0.111996	0.0173056	2E+06
17A	A. ulnaris	2.939507	0.085421	0.0169594	4E+06
17B	A. ulnaris	2.939507	0.075930	0.0162672	4E+06
17C	A. ulnaris	2.939507	0.071184	0.0159211	4E+06
17D	A. ulnaris	1.623310	0.066438	0.0155750	4E+06
18A	A. radialis	3.115000	0.080675	0.0152289	4E+06
18B	A. radialis	3.115000	0.075930	0.0148828	4E+06
18C	A. radialis	3.115000	0.071184	0.0145367	4E+06
18D	A. radialis	0.965211	0.066438	0.0141906	4E+06
19A	A. interossea volaris	3.465985	0.042710	0.0096911	8E+06
20A	A. coelica	0.481257	0.203017	0.0242981	2E+06
21A	A. gastrica sin.	3.416922	0.093700	0.0170846	2E+06
22A	A. lienalis	3.031917	0.143153	0.0205015	2E+06
23A	A. hepatica	3.176294	0.114523	0.0186032	2E+06
24A	A. renalis	1.198512	0.105331	0.0153643	2E+06
25AA	A. mesenterica sup.	2.209756	0.081024	0.0203872	2E+06
25BA	A. mesenterica inf.	1.872675	0.064819	0.0127051	2E+06

26AA	A. carotis com. sin.	2.839414	0.192606	0.0239185	1E+06
26AB	A. carotis com. sin.	2.215283	0.150269	0.0186609	1E+06
26AC	A. carotis com. sin.	2.215283	0.150269	0.0186609	1E+06
26AD	A. carotis com. sin.	1.163962	0.150269	0.0186609	1E+06
26AE	A. carotis int. sin.	3.710348	0.120400	0.0223250	8E+06
26AF	A. carotis int. sin.	3.710348	0.087749	0.0193483	8E+06
26AG	A. cerebri anterior sin.	3.710348	0.056459	0.0128989	16E+06
26AH	A. carotis ext. sin.	3.710348	0.120400	0.0223250	8E+06
26AI	A. carotis ext. sin.	3.710348	0.087749	0.0193483	8E+06
26AJ	A. carotis ext. sin.	3.710348	0.056459	0.0128989	16E+06
26BA	A. carotis com. dextra.	2.215283	0.150269	0.0186609	1E+06
26BB	A. carotis com. dextra.	2.215283	0.150269	0.0186609	1E+06
26BC	A. carotis com. dextra.	2.215283	0.150269	0.0186609	1E+06
26BD	A. carotis ext. dextra.	3.710348	0.120400	0.0223250	8E+06
26BE	A. carotis ext. dextra.	3.710348	0.087749	0.0193483	8E+06
26BF	A. carotis ext. dextra.	3.710348	0.056459	0.0128989	16E+06
26BG	A. carotis int. dextra.	3.710348	0.120400	0.0223250	8E+06
26BH	A. carotis int. dextra.	3.710348	0.087749	0.0193483	8E+06
26BI	A. cerebri anterior dextra	3.710348	0.056459	0.0128989	16E+06
27A	A. vertebralis	2.665848	0.076353	0.0136254	2E+06
27B	A. vertebralis	2.665848	0.074322	0.0133292	2E+06

		Windkessel parameters		
		Rc	Rp	Cw
Before:	2A	154723	147949	7.90717E-07
Between:	3A and 3B	154723	996818	3.02370E-06
	3B and 4A	154723	996818	3.02370E-06
	4A and 4B	154723	996818	3.02370E-06
	4B and 4C	154723	996818	3.02370E-06
At the end of:	21A	25594	426533	7.76914E-07
	22A	10151	183362	1.81167E-06
	23A	16538	286454	1.15786E-06
	24A	18648	74055	4.50604E-06
	25AA	38254	60738	4.50604E-06
	25BA	53666	298816	7.40087E-07
	26AG	209385	196093	1.30448E-06
	26AJ	209385	196093	1.30448E-06
	26BF	209385	196093	1.30448E-06
	26BI	209385	196093	1.30448E-06
	27B	39848	71263	1.73762E-06
	8B	97032	292177	7.56302E-07
	11E	234154	292177	7.56302E-07
	12E	191978	342299	6.45348E-07
	17D	77361	347665	1.16520E-06
	18D	74760	573469	1.16520E-06
	19A	250684	3028631	7.29807E-08

SUPPLEMENTAL TABLE S3 - Vascular dimensions and Windkessel parameters for 1 year of age.

2 years					
Arterial segments		Vascular dimensions			
Notation of segment	Name of artery	length	radius	wall thickness	Young's modulus
2A	Aorta ascendens	1.131027	0.944446	0.0741954	2E+06
2B	Aorta ascendens	1.131027	0.925171	0.0728382	2E+06
3A	Arcus aorta	1.131027	0.899472	0.0597182	2E+06
3B	Arcus aorta	2.205503	0.770976	0.0574562	2E+06
4A	Aorta thoracalis	2.940671	0.706728	0.0542893	2E+06
4B	Aorta thoracalis	2.940671	0.642480	0.0497652	2E+06
4C	Aorta thoracalis	2.940671	0.578232	0.0452411	2E+06
5A	Aorta abdominalis	2.997222	0.513984	0.0407170	2E+06
5B	Aorta abdominalis	2.247415	0.313139	0.0278171	2E+06
5C	Aorta abdominalis	2.247415	0.289051	0.0271386	2E+06
6A	A. iliaca communis	2.459435	0.163796	0.0237463	2E+06
7A	A. iliaca externa	2.459435	0.154161	0.0203539	2E+06
7B	A. iliaca externa	1.060101	0.144526	0.0186578	2E+06
8A	A. profundus	2.757320	0.099447	0.0182071	8E+06
8B	A. profundus femoris	2.757320	0.089503	0.0161063	8E+06
9A	A. femoralis	2.586647	0.134891	0.0179793	2E+06
9B	A. femoralis	2.669786	0.129282	0.0182071	2E+06
9C	A. femoralis	2.669786	0.119337	0.0178569	2E+06
9D	A. femoralis	2.669786	0.114364	0.0175068	2E+06
9E	A. femoralis	3.107456	0.109392	0.0171567	2E+06
10A	A. poplitea	2.757320	0.104420	0.0168065	4E+06
10B	A. poplitea	2.757320	0.099447	0.0164564	4E+06
10C	A. poplitea	2.757320	0.094475	0.0161063	4E+06
11A	A. tibialis posterior	2.932388	0.079558	0.0178569	8E+06
11B	A. tibialis posterior	2.932388	0.074586	0.0171567	8E+06
11C	A. tibialis posterior	2.932388	0.069613	0.0161063	8E+06
11D	A. tibialis posterior	2.932388	0.064641	0.0154060	8E+06
11E	A. tibialis posterior	2.319651	0.059668	0.0143556	8E+06
12A	A. tibialis anterior	3.282524	0.079558	0.0136553	8E+06
12B	A. tibialis anterior	3.282524	0.074586	0.0136553	8E+06
12C	A. tibialis anterior	3.282524	0.069613	0.0136553	8E+06
12D	A. tibialis anterior	1.881981	0.059668	0.0136553	8E+06
12E	A. tibialis anterior	3.282524	0.064641	0.0136553	8E+06
13A	A. anonyma	1.922746	0.398338	0.0389073	2E+06
14A	A. subclavia	1.922746	0.271769	0.0303115	2E+06
14B	A. subclavia	3.462167	0.233110	0.0268827	2E+06
15A	A. axillaris	3.105768	0.210551	0.0252535	2E+06
15B	A. axillaris	2.851197	0.181629	0.0232169	2E+06
16A	A. brachialis	3.207596	0.163119	0.0224023	2E+06
16B	A. brachialis	3.207596	0.153864	0.0215876	2E+06
16C	A. brachialis	3.207596	0.144609	0.0211803	2E+06
16D	A. brachialis	2.342054	0.136511	0.0203657	2E+06
17A	A. ulnaris	3.411253	0.104119	0.0199584	4E+06
17B	A. ulnaris	3.411253	0.092550	0.0191437	4E+06
17C	A. ulnaris	3.411253	0.086765	0.0187364	4E+06
17D	A. ulnaris	1.883826	0.080981	0.0183291	4E+06
18A	A. radialis	3.614910	0.098334	0.0179218	4E+06
18B	A. radialis	3.614910	0.092550	0.0175145	4E+06
18C	A. radialis	3.614910	0.086765	0.0171072	4E+06
18D	A. radialis	1.120113	0.080981	0.0166999	4E+06
19A	A. interossea volaris	4.022224	0.052059	0.0114048	8E+06
20A	A. coelica	0.565514	0.250567	0.0289543	2E+06
21A	A. gastrica sin.	4.015147	0.115646	0.0203585	2E+06
22A	A. lienalis	3.562736	0.176682	0.0244302	2E+06
23A	A. hepatica	3.732390	0.141346	0.0221681	2E+06
24A	A. renalis	1.356930	0.125256	0.0176401	2E+06
25AA	A. mesenterica sup.	2.501839	0.096350	0.0234070	2E+06
25BA	A. mesenterica inf.	2.120203	0.077080	0.0145870	2E+06

26AA	A. carotis com. sin.	3.336530	0.237718	0.0285019	1E+06
26AB	A. carotis com. sin.	2.664592	0.189844	0.0227619	1E+06
26AC	A. carotis com. sin.	2.664592	0.189844	0.0227619	1E+06
26AD	A. carotis com. sin.	1.400040	0.189844	0.0227619	1E+06
26AE	A. carotis int. sin.	4.265347	0.145376	0.0260258	8E+06
26AF	A. carotis int. sin.	4.265347	0.105952	0.0225557	8E+06
26AG	A. cerebri anterior sin.	4.265347	0.068171	0.0150372	16E+06
26AH	A. carotis ext. sin.	4.265347	0.145376	0.0260258	8E+06
26AI	A. carotis ext. sin.	4.265347	0.105952	0.0225557	8E+06
26AJ	A. carotis ext. sin.	4.265347	0.068171	0.0150372	16E+06
26BA	A. carotis com. dextra.	2.664592	0.189844	0.0227619	1E+06
26BB	A. carotis com. dextra.	2.664592	0.189844	0.0227619	1E+06
26BC	A. carotis com. dextra.	2.664592	0.189844	0.0227619	1E+06
26BD	A. carotis ext. dextra.	4.265347	0.145376	0.0260258	8E+06
26BE	A. carotis ext. dextra.	4.265347	0.105952	0.0225557	8E+06
26BF	A. carotis ext. dextra.	4.265347	0.068171	0.0150372	16E+06
26BG	A. carotis int. dextra.	4.265347	0.145376	0.0260258	8E+06
26BH	A. carotis int. dextra.	4.265347	0.105952	0.0225557	8E+06
26BI	A. cerebri anterior dextra	4.265347	0.068171	0.0150372	16E+06
27A	A. vertebralis	3.206543	0.096461	0.0166198	2E+06
27B	A. vertebralis	3.206543	0.093896	0.0162585	2E+06

		Windkessel parameters		
		Rc	Rp	Cw
Before:	2A	104251	113023	8.93286E-07
Between:	3A and 3B	104251	761505	3.41592E-06
	3B and 4A	104251	761505	3.41592E-06
	4A and 4B	104251	761505	3.41592E-06
	4B and 4C	104251	761505	3.41592E-06
At the end of:	21A	16747	325844	8.77691E-07
	22A	6613	140077	2.04667E-06
	23A	10797	218833	1.30805E-06
	24A	13101	64006	4.49937E-06
	25AA	26985	52496	4.49937E-06
	25BA	37918	258270	7.38992E-07
	26AG	144005	168805	1.30780E-06
	26AJ	144005	168805	1.30780E-06
	26BF	144005	168805	1.30780E-06
	26BI	144005	168805	1.30780E-06
	27B	24983	58861	1.81559E-06
	8B	55674	206578	9.23176E-07
	11E	135726	206578	9.23176E-07
	12E	110993	242016	7.87740E-07
	17D	52126	268033	1.30436E-06
	18D	50356	442117	1.30436E-06
	19A	170168	2334930	8.16972E-08

SUPPLEMENTAL TABLE S4 - Vascular dimensions and Windkessel parameters for 2 years of age.

5 years					
**Arterial segments**		**Vascular dimensions**			
Notation of segment	Name of artery	length	radius	wall thickness	Young's modulus
2A	Aorta ascendens	1.326896	1.130226	0.0906712	2E+06
2B	Aorta ascendens	1.326896	1.107161	0.0890126	2E+06
3A	Arcus aorta	1.326896	1.076406	0.0729793	2E+06
3B	Arcus aorta	2.587446	0.922634	0.0702149	2E+06
4A	Aorta thoracalis	3.449928	0.845748	0.0663448	2E+06
4B	Aorta thoracalis	3.449928	0.768861	0.0608160	2E+06
4C	Aorta thoracalis	3.449928	0.691975	0.0552873	2E+06
5A	Aorta abdominalis	3.516273	0.615089	0.0497586	2E+06
5B	Aorta abdominalis	2.544311	0.361617	0.0328040	2E+06
5C	Aorta abdominalis	2.544311	0.333801	0.0320039	2E+06
6A	A. iliaca communis	2.784341	0.189154	0.0280034	2E+06
7A	A. iliaca externa	2.784341	0.178027	0.0240029	2E+06
7B	A. iliaca externa	1.200147	0.166900	0.0220027	2E+06
8A	A. profundus	3.757948	0.138255	0.0258483	8E+06
8B	A. profundus femoris	3.757948	0.124430	0.0228658	8E+06
9A	A. femoralis	2.928358	0.155774	0.0212026	2E+06
9B	A. femoralis	3.638648	0.179732	0.0258483	2E+06
9C	A. femoralis	3.638648	0.165906	0.0253512	2E+06
9D	A. femoralis	3.638648	0.158994	0.0248542	2E+06
9E	A. femoralis	4.235148	0.152081	0.0243571	2E+06
10A	A. poplitea	3.757948	0.145168	0.0238600	4E+06
10B	A. poplitea	3.757948	0.138255	0.0233629	4E+06
10C	A. poplitea	3.757948	0.131342	0.0228658	4E+06
11A	A. tibialis posterior	3.996548	0.110604	0.0253512	8E+06
11B	A. tibialis posterior	3.996548	0.103691	0.0243571	8E+06
11C	A. tibialis posterior	3.996548	0.096779	0.0228658	8E+06
11D	A. tibialis posterior	3.996548	0.089866	0.0218717	8E+06
11E	A. tibialis posterior	3.161449	0.082953	0.0203804	8E+06
12A	A. tibialis anterior	4.473748	0.110604	0.0193862	8E+06
12B	A. tibialis anterior	4.473748	0.103691	0.0193862	8E+06
12C	A. tibialis anterior	4.473748	0.096779	0.0193862	8E+06
12D	A. tibialis anterior	2.564949	0.082953	0.0193862	8E+06
12E	A. tibialis anterior	4.473748	0.089866	0.0193862	8E+06
13A	A. anonyma	2.255722	0.476694	0.0475471	2E+06
14A	A. subclavia	2.255722	0.325228	0.0370425	2E+06
14B	A. subclavia	4.014547	0.275724	0.0324706	2E+06
15A	A. axillaris	3.601285	0.249041	0.0305027	2E+06
15B	A. axillaris	3.306098	0.214832	0.0280428	2E+06
16A	A. brachialis	3.719360	0.192938	0.0270588	2E+06
16B	A. brachialis	3.719360	0.181991	0.0260749	2E+06
16C	A. brachialis	3.719360	0.171044	0.0255829	2E+06
16D	A. brachialis	2.715723	0.161466	0.0245989	2E+06
17A	A. ulnaris	3.955510	0.123152	0.0241070	4E+06
17B	A. ulnaris	3.955510	0.109468	0.0231230	4E+06
17C	A. ulnaris	3.955510	0.102627	0.0226310	4E+06
17D	A. ulnaris	2.184386	0.095785	0.0221390	4E+06
18A	A. radialis	4.191659	0.116310	0.0216471	4E+06
18B	A. radialis	4.191659	0.109468	0.0211551	4E+06
18C	A. radialis	4.191659	0.102627	0.0206631	4E+06
18D	A. radialis	1.298824	0.095785	0.0201711	4E+06
19A	A. interossea volaris	4.663959	0.061576	0.0137754	8E+06
20A	A. coelica	0.663448	0.299856	0.0353839	2E+06
21A	A. gastrica sin.	4.710479	0.138395	0.0248793	2E+06
22A	A. lienalis	4.179721	0.211437	0.0298551	2E+06
23A	A. hepatica	4.378755	0.169150	0.0270908	2E+06
24A	A. renalis	1.536188	0.144647	0.0208025	2E+06
25AA	A. mesenterica sup.	2.832347	0.111267	0.0276034	2E+06
25BA	A. mesenterica inf.	2.400294	0.089013	0.0172021	2E+06

26AA	A. carotis com. sin.	3.914342	0.284479	0.0348310	1E+06
26AB	A. carotis com. sin.	3.333482	0.242264	0.0296623	1E+06
26AC	A. carotis com. sin.	3.333482	0.242264	0.0296623	1E+06
26AD	A. carotis com. sin.	1.751491	0.242264	0.0296623	1E+06
26AE	A. carotis int. sin.	4.688788	0.163013	0.0298016	8E+06
26AF	A. carotis int. sin.	4.688788	0.118806	0.0258281	8E+06
26AG	A. cerebri anterior sin.	4.688788	0.076441	0.0172187	16E+06
26AH	A. carotis ext. sin.	4.688788	0.163013	0.0298016	8E+06
26AI	A. carotis ext. sin.	4.688788	0.118806	0.0258281	8E+06
26AJ	A. carotis ext. sin.	4.688788	0.076441	0.0172187	16E+06
26BA	A. carotis com. dextra.	3.333482	0.242264	0.0296623	1E+06
26BB	A. carotis com. dextra.	3.333482	0.242264	0.0296623	1E+06
26BC	A. carotis com. dextra.	3.333482	0.242264	0.0296623	1E+06
26BD	A. carotis ext. dextra.	4.688788	0.163013	0.0298016	8E+06
26BE	A. carotis ext. dextra.	4.688788	0.118806	0.0258281	8E+06
26BF	A. carotis ext. dextra.	4.688788	0.076441	0.0172187	16E+06
26BG	A. carotis int. dextra.	4.688788	0.163013	0.0298016	8E+06
26BH	A. carotis int. dextra.	4.688788	0.118806	0.0258281	8E+06
26BI	A. cerebri anterior dextra	4.688788	0.076441	0.0172187	16E+06
27A	A. vertebralis	4.011478	0.123096	0.0216582	2E+06
27B	A. vertebralis	4.011478	0.119823	0.0211874	2E+06

		**Windkessel parameters**		
		Rc	Rp	Cw
Before:	2A	75828	64890	1.46590E-06
Between:	3A and 3B	75828	437205	5.60559E-06
	3B and 4A	75828	437205	5.60559E-06
	4A and 4B	75828	437205	5.60559E-06
	4B and 4C	75828	437205	5.60559E-06
At the end of:	21A	11877	187078	1.44031E-06
	22A	4680	80423	3.35862E-06
	23A	7648	125639	2.14653E-06
	24A	9963	44855	6.04911E-06
	25AA	20533	36789	6.04911E-06
	25BA	28919	180992	9.93526E-07
	26AG	116358	148927	1.39662E-06
	26AJ	116358	148927	1.39662E-06
	26BF	116358	148927	1.39662E-06
	26BI	116358	148927	1.39662E-06
	27B	15652	46022	2.18777E-06
	8B	29512	112030	1.60383E-06
	11E	72479	112030	1.60383E-06
	12E	59180	131249	1.36853E-06
	17D	37914	209866	1.56953E-06
	18D	36634	346171	1.56953E-06
	19A	124451	1828216	9.83058E-08

SUPPLEMENTAL TABLE S5 - Vascular dimensions and Windkessel parameters for 5 years of age.

10 years					
**Arterial segments**		**Vascular dimensions**			
Notation of segment	Name of artery	length	radius	wall thickness	Young's modulus
2A	Aorta ascendens	1.544506	1.229201	0.1125774	2E+06
2B	Aorta ascendens	1.544506	1.204116	0.1105180	2E+06
3A	Arcus aorta	1.544506	1.170668	0.0906110	2E+06
3B	Arcus aorta	3.011787	1.003430	0.0871788	2E+06
4A	Aorta thoracalis	4.015717	0.919811	0.0823737	2E+06
4B	Aorta thoracalis	4.015717	0.836191	0.0755092	2E+06
4C	Aorta thoracalis	4.015717	0.752572	0.0686447	2E+06
5A	Aorta abdominalis	4.092942	0.668953	0.0617803	2E+06
5B	Aorta abdominalis	3.373044	0.447925	0.0463882	2E+06
5C	Aorta abdominalis	3.373044	0.413469	0.0452568	2E+06
6A	A. iliaca communis	3.691256	0.234299	0.0395997	2E+06
7A	A. iliaca externa	3.691256	0.220517	0.0339426	2E+06
7B	A. iliaca externa	1.591058	0.206735	0.0311140	2E+06
8A	A. profundus	4.912375	0.168860	0.0360414	8E+06
8B	A. profundus femoris	4.912375	0.151974	0.0318828	8E+06
9A	A. femoralis	3.882183	0.192952	0.0299826	2E+06
9B	A. femoralis	4.756426	0.219518	0.0360414	2E+06
9C	A. femoralis	4.756426	0.202632	0.0353483	2E+06
9D	A. femoralis	4.756426	0.194189	0.0346552	2E+06
9E	A. femoralis	5.536168	0.185746	0.0339621	2E+06
10A	A. poplitea	4.912375	0.177303	0.0332690	4E+06
10B	A. poplitea	4.912375	0.168860	0.0325759	4E+06
10C	A. poplitea	4.912375	0.160417	0.0318828	4E+06
11A	A. tibialis posterior	5.224272	0.135088	0.0353483	8E+06
11B	A. tibialis posterior	5.224272	0.126645	0.0339621	8E+06
11C	A. tibialis posterior	5.224272	0.118202	0.0318828	8E+06
11D	A. tibialis posterior	5.224272	0.109759	0.0304966	8E+06
11E	A. tibialis posterior	4.132633	0.101316	0.0284173	8E+06
12A	A. tibialis anterior	5.848065	0.135088	0.0270311	8E+06
12B	A. tibialis anterior	5.848065	0.126645	0.0270311	8E+06
12C	A. tibialis anterior	5.848065	0.118202	0.0270311	8E+06
12D	A. tibialis anterior	3.352891	0.101316	0.0270311	8E+06
12E	A. tibialis anterior	5.848065	0.109759	0.0270311	8E+06
13A	A. anonyma	2.625661	0.518439	0.0590345	2E+06
14A	A. subclavia	2.625661	0.353709	0.0459920	2E+06
14B	A. subclavia	4.883287	0.313368	0.0421303	2E+06
15A	A. axillaris	4.380596	0.283042	0.0395770	2E+06
15B	A. axillaris	4.021530	0.244163	0.0363853	2E+06
16A	A. brachialis	4.524222	0.219280	0.0351086	2E+06
16B	A. brachialis	4.524222	0.206838	0.0338319	2E+06
16C	A. brachialis	4.524222	0.194397	0.0331936	2E+06
16D	A. brachialis	3.303400	0.183511	0.0319169	2E+06
17A	A. ulnaris	4.811474	0.139966	0.0312786	4E+06
17B	A. ulnaris	4.811474	0.124414	0.0300019	4E+06
17C	A. ulnaris	4.811474	0.116638	0.0293636	4E+06
17D	A. ulnaris	2.657083	0.108862	0.0287252	4E+06
18A	A. radialis	5.098726	0.132190	0.0280869	4E+06
18B	A. radialis	5.098726	0.124414	0.0274485	4E+06
18C	A. radialis	5.098726	0.116638	0.0268102	4E+06
18D	A. radialis	1.579887	0.108862	0.0261719	4E+06
19A	A. interossea volaris	5.673230	0.069983	0.0178735	8E+06
20A	A. coelica	0.772253	0.326115	0.0439326	2E+06
21A	A. gastrica sin.	5.482998	0.150514	0.0308901	2E+06
22A	A. lienalis	4.865195	0.229953	0.0370682	2E+06
23A	A. hepatica	5.096871	0.183962	0.0336359	2E+06
24A	A. renalis	2.036555	0.179170	0.0294169	2E+06
25AA	A. mesenterica sup.	3.754898	0.137823	0.0390340	2E+06
25BA	A. mesenterica inf.	3.182117	0.110259	0.0243255	2E+06

26AA	A. carotis com. sin.	4.556294	0.309391	0.0432462	1E+06
26AB	A. carotis com. sin.	4.225535	0.286931	0.0401068	1E+06
26AC	A. carotis com. sin.	4.225535	0.286931	0.0401068	1E+06
26AD	A. carotis com. sin.	2.220197	0.286931	0.0401068	1E+06
26AE	A. carotis int. sin.	4.951337	0.160838	0.0335684	8E+06
26AF	A. carotis int. sin.	4.951337	0.117221	0.0290926	8E+06
26AG	A. cerebri anterior sin.	4.951337	0.075421	0.0193951	16E+06
26AH	A. carotis ext. sin.	4.951337	0.160838	0.0335684	8E+06
26AI	A. carotis ext. sin.	4.951337	0.117221	0.0290926	8E+06
26AJ	A. carotis ext. sin.	4.951337	0.075421	0.0193951	16E+06
26BA	A. carotis com. dextra.	4.225535	0.286931	0.0401068	1E+06
26BB	A. carotis com. dextra.	4.225535	0.286931	0.0401068	1E+06
26BC	A. carotis com. dextra.	4.225535	0.286931	0.0401068	1E+06
26BD	A. carotis ext. dextra.	4.951337	0.160838	0.0335684	8E+06
26BE	A. carotis ext. dextra.	4.951337	0.117221	0.0290926	8E+06
26BF	A. carotis ext. dextra.	4.951337	0.075421	0.0193951	16E+06
26BG	A. carotis int. dextra.	4.951337	0.160838	0.0335684	8E+06
26BH	A. carotis int. dextra.	4.951337	0.117221	0.0290926	8E+06
26BI	A. cerebri anterior dextra	4.951337	0.075421	0.0193951	16E+06
27A	A. vertebralis	5.084966	0.145792	0.0292843	2E+06
27B	A. vertebralis	5.084966	0.141915	0.0286477	2E+06

		Windkessel parameters		
		Rc	Rp	Cw
Before:	2A	61944	36360	3.20739E-06
Between:	3A and 3B	61944	244976	1.22650E-05
	3B and 4A	61944	244976	1.22650E-05
	4A and 4B	61944	244976	1.22650E-05
	4B and 4C	61944	244976	1.22650E-05
At the end of:	21A	10595	104824	3.15139E-06
	22A	4183	45063	7.34867E-06
	23A	6829	70398	4.69662E-06
	24A	6896	24574	1.35365E-05
	25AA	14141	20155	1.35365E-05
	25BA	20031	99159	2.22328E-06
	26AG	125245	137821	1.85022E-06
	26AJ	125245	137821	1.85022E-06
	26BF	125245	137821	1.85022E-06
	26BI	125245	137821	1.85022E-06
	27B	11819	31976	3.86037E-06
	8B	20963	61666	3.57218E-06
	11E	51467	61666	3.57218E-06
	12E	42066	72245	3.04812E-06
	17D	30972	177491	2.27522E-06
	18D	29966	292768	2.27522E-06
	19A	102072	1546183	1.42506E-07

SUPPLEMENTAL TABLE S6 - Vascular dimensions and Windkessel parameters for 10 years of age.

15 years

Arterial segments		Vascular dimensions			
Notation of segment	Name of artery	length	radius	wall thickness	Young's modulus
2A	Aorta ascendens	1.855612	1.398445	0.1437069	2E+06
2B	Aorta ascendens	1.855612	1.369905	0.1410781	2E+06
3A	Arcus aorta	1.855612	1.331853	0.1156665	2E+06
3B	Arcus aorta	3.618444	1.141588	0.1112852	2E+06
4A	Aorta thoracalis	4.824592	1.046456	0.1051514	2E+06
4B	Aorta thoracalis	4.824592	0.951323	0.0963888	2E+06
4C	Aorta thoracalis	4.824592	0.856191	0.0876261	2E+06
5A	Aorta abdominalis	4.917373	0.761059	0.0788635	2E+06
5B	Aorta abdominalis	4.795013	0.602973	0.0700655	2E+06
5C	Aorta abdominalis	4.795013	0.556591	0.0683566	2E+06
6A	A. iliaca communis	5.247373	0.315401	0.0598120	2E+06
7A	A. iliaca externa	5.247373	0.296848	0.0512674	2E+06
7B	A. iliaca externa	2.261799	0.278295	0.0469951	2E+06
8A	A. profundus	5.995970	0.195173	0.0467411	8E+06
8B	A. profundus femoris	5.995970	0.175656	0.0413479	8E+06
9A	A. femoralis	5.518789	0.259742	0.0452862	2E+06
9B	A. femoralis	5.805622	0.253725	0.0467411	2E+06
9C	A. femoralis	5.805622	0.234208	0.0458422	2E+06
9D	A. femoralis	5.805622	0.224449	0.0449433	2E+06
9E	A. femoralis	6.757363	0.214690	0.0440445	2E+06
10A	A. poplitea	5.995970	0.204932	0.0431456	4E+06
10B	A. poplitea	5.995970	0.195173	0.0422467	4E+06
10C	A. poplitea	5.995970	0.185414	0.0413479	4E+06
11A	A. tibialis posterior	6.376667	0.156138	0.0458422	8E+06
11B	A. tibialis posterior	6.376667	0.146380	0.0440445	8E+06
11C	A. tibialis posterior	6.376667	0.136621	0.0413479	8E+06
11D	A. tibialis posterior	6.376667	0.126862	0.0395501	8E+06
11E	A. tibialis posterior	5.044229	0.117104	0.0368535	8E+06
12A	A. tibialis anterior	7.138060	0.156138	0.0350558	8E+06
12B	A. tibialis anterior	7.138060	0.146380	0.0350558	8E+06
12C	A. tibialis anterior	7.138060	0.136621	0.0350558	8E+06
12D	A. tibialis anterior	4.092488	0.117104	0.0350558	8E+06
12E	A. tibialis anterior	7.138060	0.126862	0.0350558	8E+06
13A	A. anonyma	3.154541	0.589820	0.0753585	2E+06
14A	A. subclavia	3.154541	0.402410	0.0587095	2E+06
14B	A. subclavia	6.246528	0.379582	0.0572598	2E+06
15A	A. axillaris	5.603503	0.342848	0.0537895	2E+06
15B	A. axillaris	5.144199	0.295754	0.0494517	2E+06
16A	A. brachialis	5.787224	0.265613	0.0477165	2E+06
16B	A. brachialis	5.787224	0.250543	0.0459814	2E+06
16C	A. brachialis	5.787224	0.235473	0.0451138	2E+06
16D	A. brachialis	4.225592	0.222286	0.0433787	2E+06
17A	A. ulnaris	6.154667	0.169540	0.0425111	4E+06
17B	A. ulnaris	6.154667	0.150703	0.0407759	4E+06
17C	A. ulnaris	6.154667	0.141284	0.0399084	4E+06
17D	A. ulnaris	3.398846	0.131865	0.0390408	4E+06
18A	A. radialis	6.522110	0.160121	0.0381732	4E+06
18B	A. radialis	6.522110	0.150703	0.0373057	4E+06
18C	A. radialis	6.522110	0.141284	0.0364381	4E+06
18D	A. radialis	2.020936	0.131865	0.0355705	4E+06
19A	A. interossea volaris	7.256996	0.084770	0.0242921	8E+06
20A	A. coelica	0.927806	0.371016	0.0560807	2E+06
21A	A. gastrica sin.	6.587424	0.171238	0.0394318	2E+06
22A	A. lienalis	5.845179	0.261614	0.0473181	2E+06
23A	A. hepatica	6.123521	0.209291	0.0429368	2E+06
24A	A. renalis	2.895102	0.241189	0.0444318	2E+06
25AA	A. mesenterica sup.	5.337845	0.185530	0.0589575	2E+06
25BA	A. mesenterica inf.	4.523597	0.148424	0.0367417	2E+06

26AA	A. carotis com. sin.	5.474057	0.351990	0.0552045	1E+06
26AB	A. carotis com. sin.	5.359684	0.344635	0.0540510	1E+06
26AC	A. carotis com. sin.	5.359684	0.344635	0.0540510	1E+06
26AD	A. carotis com. sin.	2.816105	0.344635	0.0540510	1E+06
26AE	A. carotis int. sin.	5.570030	0.171336	0.0401231	8E+06
26AF	A. carotis int. sin.	5.570030	0.124872	0.0347733	8E+06
26AG	A. cerebri anterior sin.	5.570030	0.080344	0.0231822	16E+06
26AH	A. carotis ext. sin.	5.570030	0.171336	0.0401231	8E+06
26AI	A. carotis ext. sin.	5.570030	0.124872	0.0347733	8E+06
26AJ	A. carotis ext. sin.	5.570030	0.080344	0.0231822	16E+06
26BA	A. carotis com. dextra.	5.359684	0.344635	0.0540510	1E+06
26BB	A. carotis com. dextra.	5.359684	0.344635	0.0540510	1E+06
26BC	A. carotis com. dextra.	5.359684	0.344635	0.0540510	1E+06
26BD	A. carotis ext. dextra.	5.570030	0.171336	0.0401231	8E+06
26BE	A. carotis ext. dextra.	5.570030	0.124872	0.0347733	8E+06
26BF	A. carotis ext. dextra.	5.570030	0.080344	0.0231822	16E+06
26BG	A. carotis int. dextra.	5.570030	0.171336	0.0401231	8E+06
26BH	A. carotis int. dextra.	5.570030	0.124872	0.0347733	8E+06
26BI	A. cerebri anterior dextra	5.570030	0.080344	0.0231822	16E+06
27A	A. vertebralis	6.449789	0.175112	0.0394658	2E+06
27B	A. vertebralis	6.449789	0.170455	0.0386079	2E+06

		Windkessel parameters		
		Rc	Rp	Cw
Before:	2A	44281	26019	5.27834E-06
Between:	3A and 3B	44281	175309	2.01844E-05
	3B and 4A	44281	175309	2.01844E-05
	4A and 4B	44281	175309	2.01844E-05
	4B and 4C	44281	175309	2.01844E-05
At the end of:	21A	8577	75014	5.18619E-06
	22A	3390	32248	1.20936E-05
	23A	5532	50378	7.72914E-06
	24A	4011	16665	2.35071E-05
	25AA	8188	13668	2.35071E-05
	25BA	11665	67246	3.86089E-06
	26AG	115330	139546	2.15202E-06
	26AJ	115330	139546	2.15202E-06
	26BF	115330	139546	2.15202E-06
	26BI	115330	139546	2.15202E-06
	27B	8603	25429	5.71689E-06
	8B	16446	44044	5.89004E-06
	11E	40317	44044	5.89004E-06
	12E	32994	51600	5.02593E-06
	17D	22140	125919	3.77688E-06
	18D	21447	207701	3.77688E-06
	19A	73330	1096923	2.36560E-07

SUPPLEMENTAL TABLE S7 - Vascular dimensions and Windkessel parameters for 15 years of age.

20 years						
**Arterial segments**		**Vascular dimensions**				
Notation of segment	Name of artery	length	radius	wall thickness	Young's modulus
2A	Aorta ascendens	2.0	1.470	0.164	2E+06
2B	Aorta ascendens	2.0	1.440	0.161	2E+06
3A	Arcus aorta	2.0	1.400	0.132	2E+06
3B	Arcus aorta	3.9	1.200	0.127	2E+06
4A	Aorta thoracalis	5.2	1.100	0.120	2E+06
4B	Aorta thoracalis	5.2	1.000	0.110	2E+06
4C	Aorta thoracalis	5.2	0.900	0.100	2E+06
5A	Aorta abdominalis	5.3	0.800	0.090	2E+06
5B	Aorta abdominalis	5.3	0.650	0.082	2E+06
5C	Aorta abdominalis	5.3	0.600	0.080	2E+06
6A	A. iliaca communis	5.8	0.340	0.070	2E+06
7A	A. iliaca externa	5.8	0.320	0.060	2E+06
7B	A. iliaca externa	2.5	0.300	0.055	2E+06
8A	A. profundus	6.3	0.200	0.052	8E+06
8B	A. profundus femoris	6.3	0.180	0.046	8E+06
9A	A. femoralis	6.1	0.280	0.053	2E+06
9B	A. femoralis	6.1	0.260	0.052	2E+06
9C	A. femoralis	6.1	0.240	0.051	2E+06
9D	A. femoralis	6.1	0.230	0.050	2E+06
9E	A. femoralis	7.1	0.220	0.049	2E+06
10A	A. poplitea	6.3	0.210	0.048	4E+06
10B	A. poplitea	6.3	0.200	0.047	4E+06
10C	A. poplitea	6.3	0.190	0.046	4E+06
11A	A. tibialis posterior	6.7	0.160	0.051	8E+06
11B	A. tibialis posterior	6.7	0.150	0.049	8E+06
11C	A. tibialis posterior	6.7	0.140	0.046	8E+06
11D	A. tibialis posterior	6.7	0.130	0.044	8E+06
11E	A. tibialis posterior	5.3	0.120	0.041	8E+06
12A	A. tibialis anterior	7.5	0.160	0.039	8E+06
12B	A. tibialis anterior	7.5	0.150	0.039	8E+06
12C	A. tibialis anterior	7.5	0.140	0.039	8E+06
12D	A. tibialis anterior	4.3	0.120	0.039	8E+06
12E	A. tibialis anterior	7.5	0.130	0.039	8E+06
13A	A. anonyma	3.4	0.620	0.086	2E+06
14A	A. subclavia	3.4	0.423	0.067	2E+06
14B	A. subclavia	6.8	0.403	0.066	2E+06
15A	A. axillaris	6.1	0.364	0.062	2E+06
15B	A. axillaris	5.6	0.314	0.057	2E+06
16A	A. brachialis	6.3	0.282	0.055	2E+06
16B	A. brachialis	6.3	0.266	0.053	2E+06
16C	A. brachialis	6.3	0.250	0.052	2E+06
16D	A. brachialis	4.6	0.236	0.050	2E+06
17A	A. ulnaris	6.7	0.180	0.049	4E+06
17B	A. ulnaris	6.7	0.160	0.047	4E+06
17C	A. ulnaris	6.7	0.150	0.046	4E+06
17D	A. ulnaris	3.7	0.140	0.045	4E+06
18A	A. radialis	7.1	0.170	0.044	4E+06
18B	A. radialis	7.1	0.160	0.043	4E+06
18C	A. radialis	7.1	0.150	0.042	4E+06
18D	A. radialis	2.2	0.140	0.041	4E+06
19A	A. interossea volaris	7.9	0.090	0.028	8E+06
20A	A. coelica	1.0	0.390	0.064	2E+06
21A	A. gastrica sin.	7.1	0.180	0.045	2E+06
22A	A. lienalis	6.3	0.275	0.054	2E+06
23A	A. hepatica	6.6	0.220	0.049	2E+06
24A	A. renalis	3.2	0.260	0.052	2E+06
25AA	A. mesenterica sup.	5.9	0.200	0.069	2E+06
25BA	A. mesenterica inf.	5.0	0.160	0.043	2E+06

26AA	A. carotis com. sin.	5.9	0.370	0.063	1E+06
26AB	A. carotis com. sin.	5.9	0.370	0.063	1E+06
26AC	A. carotis com. sin.	5.9	0.370	0.063	1E+06
26AD	A. carotis com. sin.	3.1	0.370	0.063	1E+06
26AE	A. carotis int. sin.	5.9	0.177	0.045	8E+06
26AF	A. carotis int. sin.	5.9	0.129	0.039	8E+06
26AG	A. cerebri anterior sin.	5.9	0.083	0.026	16E+06
26AH	A. carotis ext. sin.	5.9	0.177	0.045	8E+06
26AI	A. carotis ext. sin.	5.9	0.129	0.039	8E+06
26AJ	A. carotis ext. sin.	5.9	0.083	0.026	16E+06
26BA	A. carotis com. dextra.	5.9	0.370	0.063	1E+06
26BB	A. carotis com. dextra.	5.9	0.370	0.063	1E+06
26BC	A. carotis com. dextra.	5.9	0.370	0.063	1E+06
26BD	A. carotis ext. dextra.	5.9	0.177	0.045	8E+06
26BE	A. carotis ext. dextra.	5.9	0.129	0.039	8E+06
26BF	A. carotis ext. dextra.	5.9	0.083	0.026	16E+06
26BG	A. carotis int. dextra.	5.9	0.177	0.045	8E+06
26BH	A. carotis int. dextra.	5.9	0.129	0.039	8E+06
26BI	A. cerebri anterior dextra	5.9	0.083	0.026	16E+06
27A	A. vertebralis	7.1	0.188	0.046	2E+06
27B	A. vertebralis	7.1	0.183	0.045	2E+06

		**Windkessel parameters**		
		Rc	Rp	Cw
Before:	2A	40459	21150	7.00000E-06
Between:	3A and 3B	40459	142500	2.67680E-05
	3B and 4A	40459	142500	2.67680E-05
	4A and 4B	40459	142500	2.67680E-05
	4B and 4C	40459	142500	2.67680E-05
At the end of:	21A	8008	60975	6.87780E-06
	22A	3170	26213	1.60382E-05
	23A	5168	40950	1.02502E-05
	24A	3567	12825	3.29280E-05
	25AA	7250	10519	3.29280E-05
	25BA	10358	51750	5.40820E-06
	26AG	111303	120937	2.67680E-06
	26AJ	111303	120937	2.67680E-06
	26BF	111303	120937	2.67680E-06
	26BI	111303	120937	2.67680E-06
	27B	7705	25313	6.19100E-06
	8B	16140	35850	7.80060E-06
	11E	39471	35850	7.80060E-06
	12E	32341	42000	6.65620E-06
	17D	20230	72750	7.04700E-06
	18D	19614	120000	7.04700E-06
	19A	67107	633750	4.41380E-07

SUPPLEMENTAL TABLE S8 - Vascular dimensions and Windkessel parameters for 20 years of age.

References

1. Westerhof N, Bosman F, De Vries CJ, Noordergraaf A. Analog studies of the human systemic arterial tree. *J Biomech.* (1969) 2:121-43.
2. Hickson SS, Butlin M, Graves M, Taviani V, Avolio AP, McEniery CM, et al. The relationship of age with regional aortic stiffness and diameter. *JACC Cardiovasc Imaging.* (2010) 3:1247-55.
3. Guyton AC, Hall JE. Textbook of Medical Physiology. 9th Ed. W.B. Saunders, Philadelphia, 1996.
