# Supplementary material for: Pressure and Flow Relations in the Systemic Arterial Tree Throughout Development From Newborn to Adult
Source: Front Pediatr. 2020 May 19;8:251. doi: 10.3389/fped.2020.00251 (PMC7248228; doi:10.3389/fped.2020.00251)
Supplement: Supplementary file 2 [file Data_Sheet_2.pdf]

Supplemental material:
Descriptions of body development

Height

Height as a function of age (in months) is described by the data of Freeman et al. [1] which is reported for boys:

Height = 1.82894241483E-09*age^5 - 1.30407736046844E-06*age^4 + 0.000334537285678893*age^3 - 0.03778452806052*age^2 + 2.281919542168*age + 53

Proportions

Proportional size of body divisions as a function of age (in months) is taken from Morris and Anson [2]:

Head to Stature ratio = 0.000230548*age^2 - 0.008082108*age + 0.242037160

Thorax to Stature ratio = 0.000154047*age^2 - 0.004977775*age + 0.276756199

Abdomen to Stature ratio = -0.00003492*age^3 + 0.00126474*age^2 - 0.0109103*age + 0.149864

Femur&Tibia to Stature ratio = 0.000034920*age^3 - 0.001649333*age^2 + 0.023970210*age + 0.331343089

Arm length is equal to thorax + abdomen length (based on Morris and Anson [2])

Radii are taken to grow proportional with the increase in segmental stature length.

Weight

Neonatal weight until 1 year of age (for boys; for girls a factor 0.92 can be applied) is based on the report by Wright et al. [3]:

Weight = -0.0351*age^2 + 0.9413*age + 3.5 (age < 12 months)

After 1 year of age the weight (for boys; for girls the weight is the same up to 14 years, after which it increases slower) is given by Burmaster et al. [4]:

Weight = -4 10^-6*age^3 + 0.0021*age^2 + 0.0158*age + 9.25611 (age > 12 months)

Volumes

From the stature, based on Freeman et al. [1] and the proportions, derived from Morris and Anson [2], the dimensions of a certain body division, described by a cone shape (or a sphere in case of the head), can be derived. With the length and diameters, the volume of the cone is known (length and diameter are assumed the same in case of the head).

Volumes are described as follows:

```
Head = -0.0000000004*age^6 + 0.0000003189*age^5 -
0.0000946392*age^4 + 0.0135138043*age^3 - 0.9481451046*age^2 +
32.9240075809*age + 268.6998973594
```

```
Neck = -0.0000000005*age^6 + 0.0000003928*age^5 -
0.0001167738*age^4 + 0.0174422303*age^3 - 1.3250532707*age^2 +
53.2689562968*age + 194.0001468519
```

```
Thorax = -0.0000000011*age^6 + 0.0000009143*age^5 -
0.0002901987*age^4 + 0.0434968065*age^3 - 2.7522753416*age^2 +
132.4535876270*age + 973.0026191697
```

```
Abdomen = -0.0000000009*age^6 + 0.0000007445*age^5 -
0.0002278272*age^4 + 0.0331266271*age^3 - 2.0594662008*age^2 +
72.1039600093*age + 809.9981431887
```

```
Leg = -0.0000000006*age^6 + 0.0000005082*age^5 -
0.0001618973*age^4 + 0.0244683520*age^3 - 1.5955778306*age^2 +
77.8713415614*age + 269.6982505769
```

```
Arm = -0.0000000005*age^6 + 0.0000003928*age^5 -
0.0001167738*age^4 + 0.0174422303*age^3 - 1.3250532707*age^2 +
53.2689562968*age + 194.0001468519
```

Cardiac output

Cardiac output (CO) was taken from Wiesener ed. [6]:

```
CO = 0.5639850929 + 0.0185883885*Age L/min
```

Heart rate

Heart rate (HR) was taken from Wiesener ed. [5]:

```
HR = 175 Weight^-0.2 beats per minute
```

Ejection time

Based on Wilkinson et al. [6], time of end-ejection (ejt) was described as a function of heart rate:

```
ejt = (397 - 1.26*HR)/1000 s
```

This relation is based on adults undergoing a pacing protocol [6], however it is remarkably similar to relations found by Cantor et al. [7]. A group of 253 children was investigated (138 males and 115 females), their ages ranging from 0-13 years. They found that ejection time is a function of HR (and not a function of age):

```
Male:     ejt = (364 - 1.12*HR)/1000 s

Female:   ejt = (381 - 1.24*HR)/1000 s
```

Mean arterial pressure (to correct vascular wall thickness)

Mean arterial pressure (MAP), used to correct vascular wall thickness, is described as a function of age (from 0 to 20 years) based on Jackson et al. [8]:

```
MAP = 70 + Age/12 mmHg
```

References

1. Freeman JV, Cole TJ, Chinn S, Jones PR, White EM, Preece MA. Cross sectional stature and weight reference curves for the UK, 1990. *Arch Dis Child.* (1995) 73:17-24.
2. Morris H, Anson BJ. Human Anatomy: A Complete Systematic Treatise. Ed. by Barry J. Anson with Eighteen Contributors. McGraw-Hill, 1966.
3. Wright CM, Corbett SS, Drewett RF. Sex differences in weight in infancy and the British 1990 national growth standards. *BMJ.* (1996) 313:513-4.
4. Burmaster DE, Crouch EA. Lognormal distributions for body weight as a function of age for males and females in the United States, 1976-1980. *Risk Anal.* (1997) 17:499-505.
5. Wiesener H. Einführung in Die Entwicklungsphysiologie Des Kindes. Springer, 1964.
6. Wilkinson IB, MacCallum H, Flint L, Cockcroft JR, Newby DE, Webb DJ. The influence of heart rate on augmentation index and central arterial pressure in humans. *J Physiol.* (2000) 525 Pt 1:263-70.
7. Cantor A, Wanderman KL, Karolevitch T, Ovsyshcher I, Gueron M. Systolic time intervals in children: normal standards for clinical use. *Circulation.* (1978) 58:1123-9.
8. Jackson LV, Thalange NK, Cole TJ. Blood pressure centiles for Great Britain. *Arch Dis Child.* (2007) 92:298-303.
