# Supplementary material for: Pressure and Flow Relations in the Systemic Arterial Tree Throughout Development From Newborn to Adult
Source: Front Pediatr. 2020 May 19;8:251. doi: 10.3389/fped.2020.00251 (PMC7248228; doi:10.3389/fped.2020.00251)
Supplement: Supplementary file 3 [file Data_Sheet_3.pdf]

Supplemental material:
Transfer functions as function of harmonics

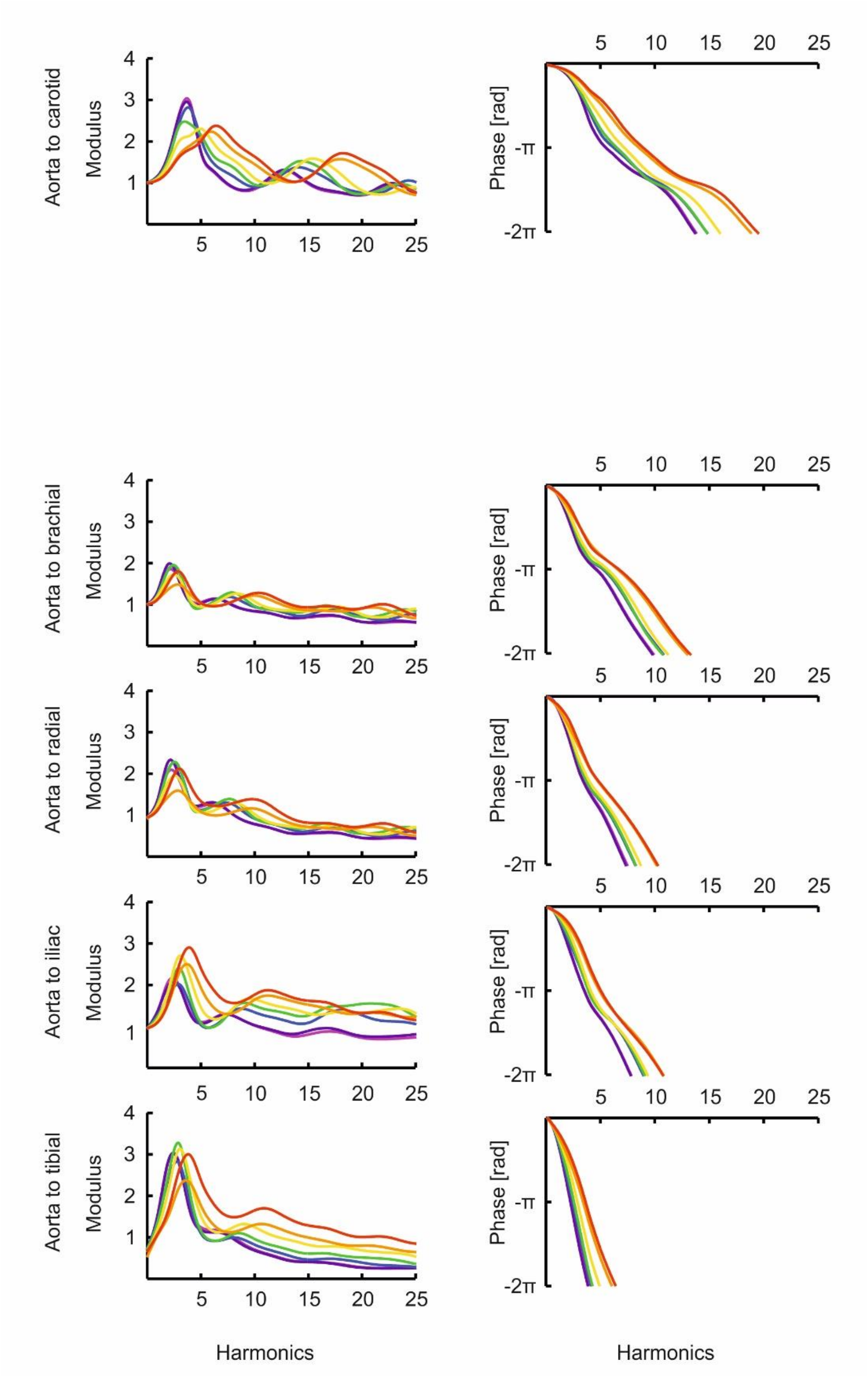

SUPPLEMENTAL FIGURE

The transfer functions between aorta and the carotid, brachial, radial, femoral and tibial artery as a function of harmonics (red, orange, yellow, green, blue, purple, violet: 0, 1, 2, 5, 10, 15, 20 years).

In the Supplemental Figure, the transfer functions are plotted as a function of harmonics as opposed to Figure 4, where the transfer functions are plotted as a function of frequency. The harmonics are related to the frequency through a factor that is the reciprocated value of the heart rate. E.g., for 0 years of age, heart rate is 136 beats per minute so the first harmonic is at 2.27 Hz. Thus, the transfer function of a 0 year old in Figure 4, “compressed” by a factor 1/2.27 gives the harmonics. Heart rate for a 20-years old is 73 beats per minute and the first harmonic is 1.22 Hz, so a “compression” of the transfer function in Figure 3 by a factor 1/1.22 gives the spectrum as a function of harmonics shown here.

In this representation, the peaks of the transfer functions for the different ages are much closer to each other, however, the peaks are still at somewhat higher harmonics for the younger ages. Thus, the main (lowest) harmonics of the pressures of the youngest are less influenced by the transfer functions, and peripheral pressures resemble central pressures.
